# Opponent Learning with Different Representations in the Cortico-Basal Ganglia Circuits

**DOI:** 10.1523/ENEURO.0422-22.2023

**Published:** 2023-01-25

**Authors:** Kenji Morita, Kanji Shimomura, Yasuo Kawaguchi

**Affiliations:** 1 Physical and Health Education, Graduate School of Education, The University of Tokyo, Tokyo 113-0033, Japan; 2 International Research Center for Neurointelligence (WPI-IRCN), The University of Tokyo, Tokyo 113-0033, Japan; 3 Department of Behavioral Medicine, National Institute of Mental Health, National Center of Neurology and Psychiatry, Kodaira 187-8551, Japan; 4 Brain Science Institute, Tamagawa University, Machida 194-8610, Japan; 5 National Institute for Physiological Sciences (NIPS), Okazaki 444-8787, Japan

## Abstract

The direct and indirect pathways of the basal ganglia (BG) have been suggested to learn mainly from positive and negative feedbacks, respectively. Since these pathways unevenly receive inputs from different cortical neuron types and/or regions, they may preferentially use different state/action representations. We explored whether such a combined use of different representations, coupled with different learning rates from positive and negative reward prediction errors (RPEs), has computational benefits. We modeled animal as an agent equipped with two learning systems, each of which adopted individual representation (IR) or successor representation (SR) of states. With varying the combination of IR or SR and also the learning rates from positive and negative RPEs in each system, we examined how the agent performed in a dynamic reward navigation task. We found that combination of SR-based system learning mainly from positive RPEs and IR-based system learning mainly from negative RPEs could achieve a good performance in the task, as compared with other combinations. In such a combination of appetitive SR-based and aversive IR-based systems, both systems show activities of comparable magnitudes with opposite signs, consistent with the suggested profiles of the two BG pathways. Moreover, the architecture of such a combination provides a novel coherent explanation for the functional significance and underlying mechanism of diverse findings about the cortico-BG circuits. These results suggest that particularly combining different representations with appetitive and aversive learning could be an effective learning strategy in certain dynamic environments, and it might actually be implemented in the cortico-BG circuits.

## Significance Statement

Animals can learn the value of states/actions from both positive and negative feedbacks. For learning, animals need to represent each state/action, individually (like representing a person by her/his identity only) or in a relation-based manner (like representing a person by friends or descendants). Different brain circuits may learn from positive and negative feedbacks with different rates, and may represent states/actions in different ways. We explored what combination of the feedback valence-dependent learning rates and the ways of state representation performs well in a dynamic reward navigation task. We found that a particular combination performed well, and we propose that several known anatomic and physiological properties of the cortico-basal ganglia circuits may indicate implementation of such a combination.

## Introduction

In the standard reinforcement learning (RL), updates based on positive reward prediction errors (RPEs) and those based on negative RPEs are executed in the same manner. However, in the brain, there appear to exist distinct neural circuits that are specialized for appetitive or aversive learning. Specifically, a number of findings have suggested or appear to be in line with that the direct and indirect pathways of the basal ganglia (BG), originating from the striatal projection neurons (SPNs) expressing D1-type and D2-type dopamine (DA) receptors (D1Rs and D2Rs), are potentiated by positive and negative feedbacks, respectively (Frank et al., 2004; Hikida et al., 2010; Kravitz et al., 2012; Tai et al., 2012; Kim et al., 2017; Iino et al., 2020; Lee et al., 2021). There are also studies suggesting that distinct circuits involving the orbitofrontal cortex (OFC) operate for appetitive and aversive feedback-based learning (Groman et al., 2019b). Computational works suggest that dual learning systems can realize estimation of costs and benefits (Collins and Frank, 2014; Möller and Bogacz, 2019), as well as estimation of not only the mean but also the uncertainty of rewards (Mikhael and Bogacz, 2016).

These existing dual learning-system models assume that both systems use the same way of representation of states or actions. Theoretically, various ways of representation can be considered, and different brain regions or neural populations may generally use different representations (Chen et al., 2014; Town et al., 2017; Wang et al., 2020). While there is evidence that the two BG pathways receive inputs from the same types of corticostriatal neurons (Ballion et al., 2008; Kress et al., 2013), it has also been suggested that different neuron types (Lei et al., 2004; Reiner et al., 2010; Morita, 2014) and/or cortical areas (Wall et al., 2013; Lu et al., 2021) may not evenly target/activate these pathways. As for the suggested distinct OFC circuits for appetitive and aversive learning, the suggested circuits are the amygdala-OFC and OFC-nucleus accumbens (NAc) pathways, respectively (Groman et al., 2019b). Therefore, in both cases, it is conceivable that, between the appetitive and aversive learning systems, states/actions are represented by at least partially different neural regions or populations, and thus in different styles.

There are two largely different ways of state/action representation (Sutton and Barto, 1998). One is to represent each individual state/action separately. This simplest representation (or equivalent ones) has been explicitly or implicitly assumed in many previous neuroscience studies using the RL framework. The other is to represent each state/action by a set of features (e.g., represent a point in a two-dimensional space by a set of *x*- and *y*-coordinates). Among various ways of feature-based representations, recent studies (Garvert et al., 2017; Momennejad et al., 2017; Russek et al., 2017, 2021; Stachenfeld et al., 2017) suggest that representation of states by their successors, named the successor representation (SR; Dayan, 1993), may be used in the brain. SR contains information about state transitions in the environment under a given policy, and thereby enables the agent to quickly adapt to changes in distant outcomes through temporal-difference (TD) RPE-based learning, without using an explicit model of the environment. It can thus beautifully explain (Russek et al., 2017) the empirical suggestions that both (apparently) model-based behavior and model-free or habitual behavior are controlled by the DA-cortico-BG systems, although different portions appear to be responsible (Balleine and O’Doherty, 2010; Dolan and Dayan, 2013), by assuming that SR and individual (punctate) representation (IR) are respectively used.

Given these suggestions and considerations, it seems possible that there exist two neural systems, which may differently learn from positive and negative RPEs and may adopt different ways of state representations. In the present study, we examined its possible consequences through simulations. Specifically, we modeled animal as an agent equipped with two learning systems, and simulated its behavior in reward learning tasks in dynamic environments, with varying the adopted representation, SR or IR, and the learning rates for positive and negative TD-RPEs in each system.

## Materials and Methods

### Simulated reward navigation task

**Figure 1.**
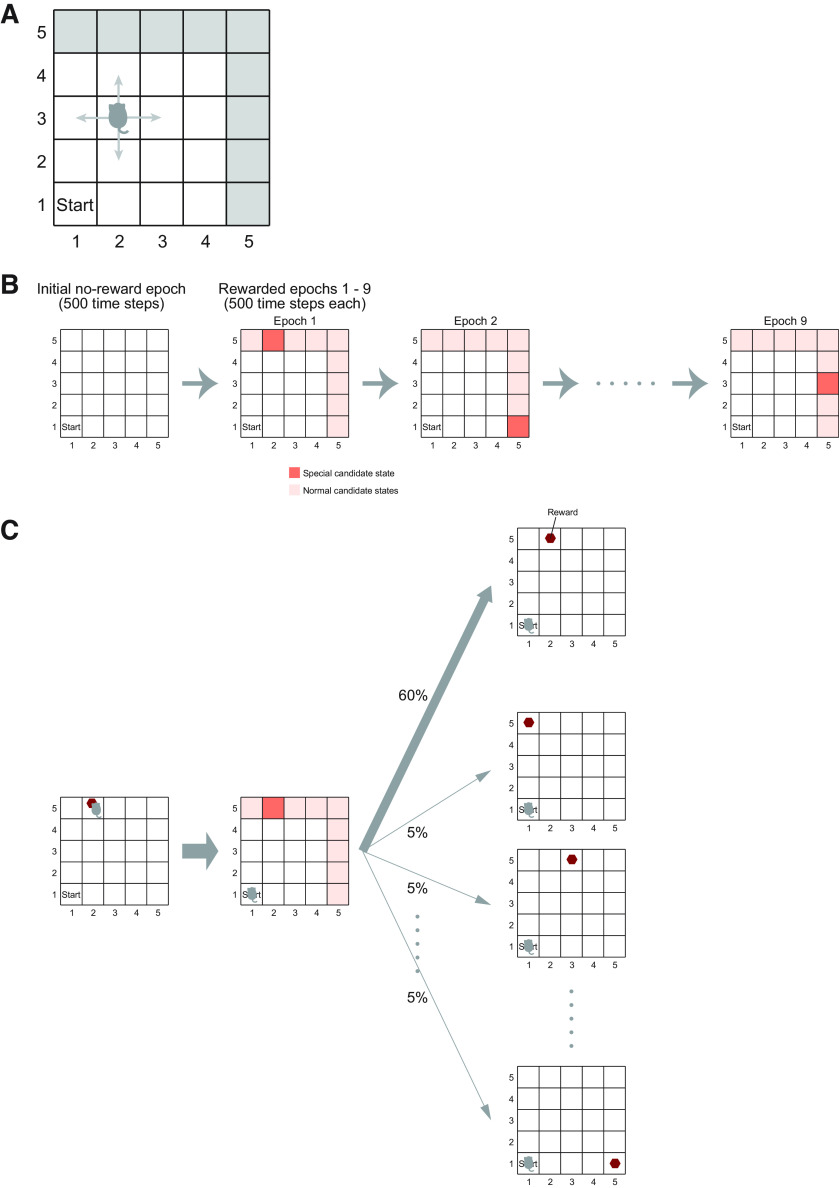
The simulated reward navigation task. ***A***, The 5 × 5 grid space, where the agent moved around. The agent started from the fixed start state, (1, 1), and moved to one of the neighboring states (two states at the corners, three states at the edges, and four states elsewhere) at each time step. There were nine reward candidate states, where reward could potentially be placed, namely, (1, 5), (2, 5), (3, 5), (4, 5), (5, 1), (5, 2), (5, 3), (5, 4), and (5, 5) (indicated by the gray color). ***B***, Epochs in the task. During the initial 500 time steps, there was no reward, and this was called the no-reward epoch. During the next 500 time steps, one of the nine reward candidate states was specified as the special candidate state, whereas the remaining eight reward candidate states were regarded as normal candidate states. There were in total nine rewarded epochs (500 time steps for each), and each one of the nine reward candidate states became the special candidate state in one of the nine epochs; the order was determined by pseudorandom permutation in each single simulation. ***C***, In the rewarded epochs, if the agent reached the rewarded state and obtained the reward, the agent was carried back to the start state, and a new reward was introduced into a state, which was the special reward candidate state with 60% probability and one of the eight normal candidate states with 5% probability for each.

We simulated a reward navigation task in a dynamic reward environment. An agent was moving around in a 5 × 5 grid space (Fig. 1*A*). The agent started from the fixed start state, which was location (1, 1), and moved to one of the neighboring states (two states at the corners, three states at the edges, and four states elsewhere) at each time step. During the initial 500 time steps, there was no reward, and the agent just moved around (Fig. 1*B*). After that, a reward (always size 1) was introduced into one of the states (locations) in the space. There were nine reward candidate states, where reward could potentially be placed, namely, (1, 5), (2, 5), (3, 5), (4, 5), (5, 1), (5, 2), (5, 3), (5, 4), and (5, 5) (i.e., the states on the two edges apart from the start state). During the next 500 time steps following the initial no-reward epoch, one of these nine candidate states was specified as the special reward candidate state, whereas the remaining eight candidate states were regarded as normal candidate states. There were in total nine rewarded epochs, each of which lasted for 500 time steps, and each one of the nine candidate states became the special candidate state in one of the nine epochs; the order was determined by pseudorandom permutation in each single simulation.

At the beginning of the first rewarded epoch, a reward was introduced into a state, which was the special reward candidate state with 60% probability and one of the eight normal reward candidate states with an equal probability (i.e., 5%) each. After that, when the agent reached the rewarded state and obtained the reward, the agent was carried back to the start state at the next time step, and a new reward was introduced into the special reward candidate state (60%) or one of the eight normal reward candidate states (5% each; Fig. 1*C*). Once reward was placed, it remained there until it was obtained by the agent even after the end of the epoch in which the reward was placed. Our motivation for setting up the two types of reward candidate states (i.e., special and normal) was to simulate the complex nature of real environments for animals in the simple grid world.

We later examined variations of the task, including those where the complicated structure of the reward candidate states was simplified. Specific motivations for considering these variations are described, one by one, in Results, Dependence on task properties. In all the variations, there was the initial 500 time-steps no-reward epoch. In the first variation, the probability that reward was placed at the special candidate state, which was 60% in the original task, was varied to 70, 80, 90, or 100%. In the second variation, periodic resets of reward placement were introduced. Specifically, reward location (state) was reset at every 500, 250, 100, or 50 time steps in the rewarded epochs; at the reset timings, reward was placed at the special candidate state for the epoch with 60% and placed at one of the other (normal) candidate states with 5% each. In the third variation, the original nine rewarded epochs with different placements of special reward candidate state (500 time steps each) were abolished and instead there was only a single rewarded epoch with 4500 time steps. For this task variation, the special candidate state was varied only across simulations. In the fourth variation, reward was always placed at the special candidate state in each epoch and the duration of rewarded epoch was shortened from 500 time steps to 100 time steps while the number of rewarded epochs was increased from 9 to 45. The order of special reward candidate states was determined by five consecutive pseudorandom permutations of the nine candidate states. In the fifth variation, the probability of reward placement at the special candidate state was changed to 1/9 (11.11...%) so that reward was placed at each of the nine candidate states with equal probability (1/9). In the sixth variation, rewarded state was determined by a fixed order, namely, (5, 1), (5, 5), (1, 5), and again (5, 1), and these were repeated throughout the task.

As a measure of performance, we examined the mean total obtained rewards averaged across simulations. Since the total duration of the rewarded epochs was fixed at 4500 time steps, considering the mean total rewards was equivalent to considering the mean reward rate per unit time step. For the original task (Fig. 1), we also examined the mean learning curve. Specifically, we calculated the mean time (number of time steps) used for obtaining the first, second, third… reward placed in each of the second to the ninth rewarded epochs, and calculated their averages across simulations (only those in which the corresponding reward was obtained) and also across the eight rewarded epochs. If a reward was placed in the second epoch and obtained in the third epoch, for example, it was regarded as the last reward in the second epoch rather than the first reward in the third epoch. The first rewarded epoch was omitted from this analysis, because there was no preceding reward and so the situation was qualitatively different from the subsequent epochs. The time used for obtaining each reward was more specifically calculated as the difference between the time steps for two consecutive goal reaches. How many rewards were obtained in a rewarded epoch depended on the type of agent and also varied from simulation to simulation. It could occur that a particular agent obtained, for example, the seventh reward placed in a rewarded epoch only in a few simulations out of a total of 100 simulations. For such a case, the mean time used for obtaining the seventh reward averaged across only the few simulations where the seventh reward was obtained would not represent the general average behavior of the agent. Therefore, to see the general average behavior, only the cases in which reward was obtained in not smaller than a quarter of a total of 100 simulations in all of the eight epochs were presented in Figure 5*A*.

### Model agent equipped with two learning systems

**Figure 2.**
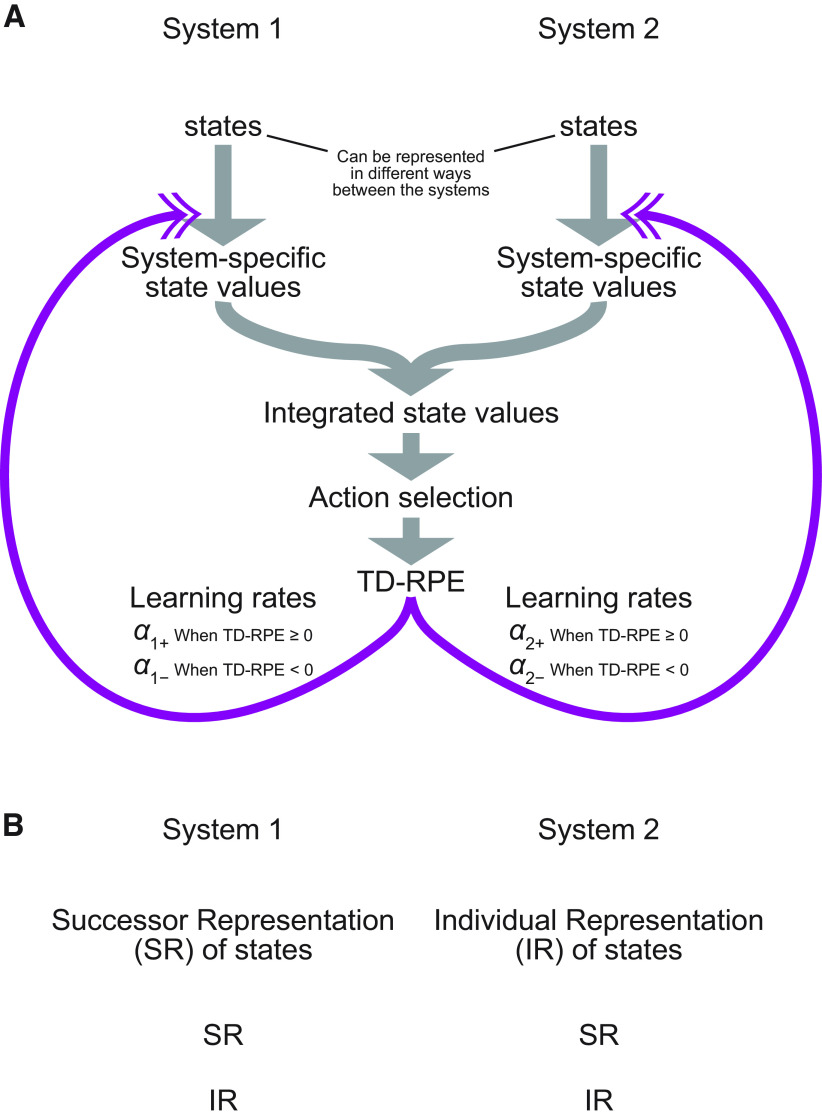
The reinforcement learning model with two systems. ***A***, Model architecture. The model consists of two learning systems, system 1 and system 2, which may use different ways of state representations [successor representation (SR) or individual representation (IR)]. Each system has its own system-specific value of each state, and their mean (average) becomes the integrated state value. The agent selects an action to move to a neighboring state depending on their integrated values in a soft-max manner. The integrated values are also used for calculating the temporal-difference reward prediction errors (TD-RPEs). The TD-RPEs were then used for updates of the system-specific state values in each of the two systems, or more precisely, updates of the system-specific values for IR-based system(s) and updates of the weights for the system-specific values for SR-based system(s). The leaning rates of the updates can differ between the two systems and also depending on whether the TD-RPE is positive (non-negative) or negative. ***B***, Possible combinations of the ways of state representation in the two systems.

The agent’s behavior was controlled by an RL model consisting of two learning systems (Fig. 2). The two systems may use different ways of state representation. We considered the successor representation (SR) and the individual representation (IR; also called the “punctate” representation), and examined the cases where system 1 and 2 used the SR and IR, respectively. We also examined the cases where only the SR or IR was used in both systems, including the cases that were equivalent to having only a single (SR-based or IR-based) system. Each of the two systems had its own system-specific value of each state (detailed below). The mean (average) of the system-specific values of the two systems was calculated and used as the integrated value of each state *S*, denoted as *V*(*S*). At any states other than the rewarded state, the agent selected an action to move to one of the neighboring states depending on their integrated state values in a soft-max manner. Specifically, the agent selected the action to move to state *S_i_*, among the neighboring states *S_j_*, with probability

$$\text{exp}\left(\beta V\left(S_{i}\right)\right)/\Sigma_{j}\left\{\text{exp}\left(\beta V\left(S_{j}\right)\right)\right\},$$

where *β* indicates the inverse temperature parameter, representing the degree of exploitation over exploration. When the agent was at state *S*(*t*) at time-step *t* and moved to *S*(*t* + 1) at *t* + 1, where *S*(*t*) was not the rewarded state, TD-RPE was calculated based on the integrated state values:

δ(t+1)=0+γV(S(t+1))−V(S(t)),

where *γ* indicates the time discount factor. When the agent was at *S*(*t*), which was the rewarded state, TD-RPE

δ(t+1)=1+0−V(S(t))

was calculated, again using the integrated state value.

In the system using the IR, the system-specific state value, *V_system_*_-_*_i_*(*S*(*t*)), where *i* was 1 or 2, was directly updated based on the TD-RPE *δ*(*t* + 1), with potentially different learning rates depending on whether the TD-RPE was positive (non-negative) or negative:

$$V_{system}{}_{-i}\left(S\left(t\right)\right)\leftarrow V_{system}{}_{-i}\left(S\left(t\right)\right)+\;\alpha_{i}{}_{+}\delta \left(t+\text{1}\right)\;\;\text{if}\;\delta \left(t+\text{1}\right)\geq 0,$$

or

$$V_{system}{}_{-i}\left(S\left(t\right)\right)\leftarrow V_{system}{}_{-i}\left(S\left(t\right)\right)+\alpha_{i}{}_{-}\delta \left(t+\text{1}\right)\;\;\text{if}\;\delta \left(t+\text{1}\right)\;<\;0,$$

where *α_i_*_+_ and *α_i_*_−_ indicate the learning rates of the system *i* for positive (non-negative) and negative TD-RPEs, respectively, and they were systematically varied as described in Results. The initial values of the system-specific state values of the system using the IR were set to 0.

In the system using the SR, the system-specific state value, *V_system_*_-_*_i_*(*S*(*t*)), where *i* was 1 or 2, was calculated as a linear function of the feature variables of the state. In the SR, every state *S_i_* was represented by a set of feature variables, which were estimated cumulative discounted future occupancies of all the states *S_j_* (*j *=* *1, …, 25), denoted as *σ*(*S_i_*, *S_j_*). These feature variables were updated through TD learning of SR features (Gershman et al., 2012; Gardner et al., 2018). Specifically, when the agent was at *S*(*t*) at *t* and moved to *S*(*t* + 1) at *t* + 1, where *S*(*t*) was not the rewarded state, the TD errors for SR features were calculated as:

$$\delta_{\text{SR}j}\left(t+\text{1}\right)=\text{1}+\gamma \sigma \left(S\left(t+\text{1}\right),S_{j}\right)-\sigma \left(S\left(t\right),S_{j}\right)\;\;\text{if}\;S\left(t\right)\text{ was}\;S_{j},$$

or

$$\delta_{\text{SR}j}\left(t+\text{1}\right)=0+\gamma \sigma \left(S\left(t+\text{1}\right),S_{j}\right)-\sigma \left(S\left(t\right),S_{j}\right)\;\;\text{if}\;S\left(t\right)\text{ was not}\;S_{j},$$

for all the states *S_j_* (*j *=* *1, …, 25). If *S*(*t*) was the rewarded state, the term *γσ*(*S*(*t* + 1), *S_j_*) was dropped. Using these TD errors, *σ*(*S*(*t*), *S_j_*) (*j *=* *1, …, 25) were updated as:

$$\sigma \left(S\left(t\right),S_{j}\right)\leftarrow \sigma \left(S\left(t\right),S_{j}\right)+\alpha_{\text{SRfeature}}\delta_{\text{SR}j}\left(t+\text{1}\right),$$

where *α*_SRfeature_ indicates the learning rate for the update of SR features and was set to 0.05 unless otherwise mentioned; *α*_SRfeature_ = 0.1, 0.15, 0.2, and 0.25 were also examined in Figures 7 and 8 and Extended Data Figures 7-1 and 8-1. The initial values of all the feature variables were set to 0. The system-specific state value, *V_system_*_-_*_i_*(*S*(*t*)), was given as a linear function of the feature variables as:

$$V_{system}{}_{-i}\left(S\left(t\right)\right)=\Sigma_{j}\left\{w_{j}\sigma \left(S\left(t\right),S_{j}\right)\right\}.$$

The weights (coefficients) *w_j_* (*j *=* *1, …, 25) were updated based on the TD-RPE *δ*(*t* + 1) described above, with potentially different learning rates depending on whether the TD-RPE was positive (non-negative) or negative:

$$w_{j}\leftarrow w_{j}+\alpha_{i}{}_{+}\sigma \left(S\left(t\right),S_{j}\right)\delta \left(t+\text{1}\right)\;\;\text{if}\;\delta \left(t+\text{1}\right)\geq 0,$$

or

$$w_{j}\leftarrow w_{j}+\alpha_{i}{}_{-}\sigma \left(S\left(t\right),S_{j}\right)\delta \left(t+\text{1}\right)\;\;\text{if}\;\delta \left(t+\text{1}\right)<0,$$

where *α_i_*_+_ and *α_i_*_−_ indicate the learning rates of the system *i* for positive (non-negative) and negative TD-RPEs, respectively, and they were systematically varied as described in Results. The initial values of the weights *w_j_* were set to 0.

### Simulated two-stage tasks

We also simulated the two-stage task (Daw et al., 2011) and its variants. In our simulation of the original two-stage task (Fig. 10*A*), there were two choice options at the first stage. Selection of one of them led to each of two pairs of second-stage options with fixed probabilities (70% or 30%), whereas selection of the other first-stage option led to each pair of second-stage options with the opposite probabilities (i.e., 30% and 70%). Then, selection of one of the second-stage options led to reward or no-reward outcome. The probability of reward for each second-stage option was independently set according to Gaussian random walk with reflecting boundaries at 0.25 and 0.75. More specifically, the reward probabilities for the four second-stage options were independently changed at each trial by adding pseudo normal random numbers with mean 0 and SD 0.025, and reflected at 0.25 and 0.75, throughout the task consisting of 201 trials (Fig. 10*B*, left).

We also simulated a variant of the task, where the probabilities of reward for the four second-stage options (two options for each of the two pairs) were set to specific values and the option-probability contingency was changed three times during the task consisting of 201 trials. Specifically, the probabilities were initially set to (0.1 and 0.5) for the first and second option of the first pair and (0.5 and 0.9) for the first and second option of the second pair, respectively, and changed to (0.9 and 0.5) and (0.5 and 0.1) at the 51th trial, (0.5 and 0.1) and (0.9 and 0.5) at the 101th trial, and (0.5 and 0.9) and (0.1 and 0.5) at the 151th trial (Fig. 10*C*, left).

We further simulated another variant of the task, in which there were three, rather than two, first-stage options and three pairs of second-stage options (Fig. 10*D*, left). Selection of each of the three first-stage options lead to one of three pairs of second-stage options with fixed probabilities: (60%, 20%, 20%), (20%, 60%, 20%), and (20%, 20%, 60%). The probabilities of reward for the six second-stage options (two options for each of the three pairs) were set to specific values and the option-probability contingency was changed two times during the task consisting of 150 trials. Specifically, the probabilities were initially set to (0.5, 0.9), (0.1, 0.5), and (0.1, 0.5) for the (first, second) option of the first, second, and third pair, and changed to (0.5, 0.1), (0.9, 0.5), and (0.5, 0.1) at the 51th trial, and (0.1, 0.5), (0.1, 0.5), and (0.5, 0.9) at the 101th trial (Fig. 10*D*, right).

In all the variants of the two-stage tasks, at every trial, SARSA-type TD RPE for the first stage was calculated after the second-stage choice was determined:

$$\delta_{k}\left(\text{1}\right)=0+\gamma V\left(O_{k}\left(\text{2}\right)\right)-V\left(O_{k}\left(\text{1}\right)\right),$$

where *O_k_*(1) and *O_k_*(2) represent the chosen option for the first and second stage at the *k*-th trial, respectively. *V*(*O*) represents the value of option *O*. *γ* Is the time discount factor, which was assumed to be 1 (i.e., no temporal discounting) in the two-stage tasks. Then TD RPE for the second stage:

$$\delta_{k}\left(\text{2}\right)=R+0-V\left(O_{k}\left(\text{2}\right)\right)$$

was calculated after reward (*R *=* *1 or 0) was determined, where *V*(*O_k_*(2)) reflected *δ_k_*(1)-based updates of the weights of the SR-based system-specific values in the cases with SR-based system(s) (see below). The IR-based system(s) learned the value of each first-stage and second-stage option (in total 2 + 4 = 6 options or 3 + 6 = 9 options) through RPE-based updates, with the initial values set to 0. The SR-based system(s) had the SR of the first-stage and second-stage options, and leaned their system-specific values through RPE-based updates of their weights. The SR matrix (6 × 6 or 9 × 9) was initialized to the one under the random policy regarding the choice at the second stage, incorporating the presumed stage-transition probabilities. The SR matrix was then updated by using the prediction errors of SR features. More specifically, at every trial, the SR features for the option chosen at the first stage was updated according to the prediction errors after the stage transition occurred and the second-stage option was determined, with the learning rate set to 0.05. Choice at both stages was made in the soft-max manner with the degree of exploitation over exploration (inverse temperature; *β*) set to 5.

### Simulations and statistics

**Figure 3.**
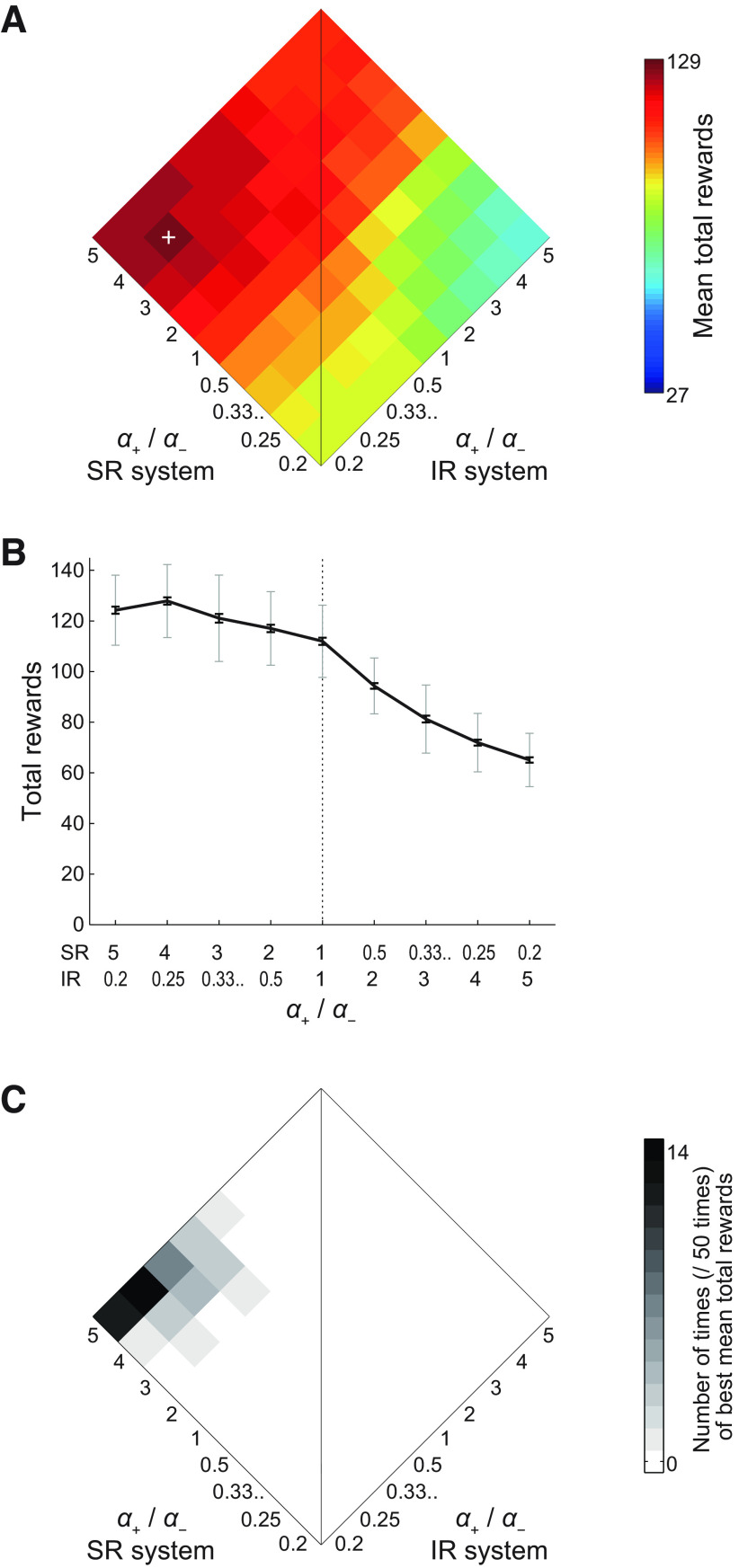
Performance of the model consisting of a system using the SR and another system using the IR, and its dependence on the ratios of the learning rates from positive and negative TD-RPEs in each system. ***A***, Mean performance over *n *=* *100 simulations for each condition. The axis rising to the left indicates the ratio of positive-/negative-error-based learning rates (denoted as *α*_+_/*α*_−_) in the system using the SR, while the axis rising to the right indicates the same ratio in the system using the IR. The sum of the learning rates from positive and negative TD-RPEs (*α*_+_ + *α*_−_) in each system was constant at 1 in any conditions shown in this figure. The inverse temperature *β* was 5, and the time discount factor *γ* was 0.7. The vertical line corresponds to conditions where the *α*_+_/*α*_−_ ratio is equal for both systems (bottom: negative error-based learning dominates; top: positive error-based learning dominates). The left side of the vertical line corresponds to conditions where the *α*_+_/*α*_−_ ratio is larger in the SR-based system than in the IR-based system, whereas the opposite is the case for the right side of the vertical line. The color in each square pixel indicates the mean total obtained rewards in the task, averaged across 100 simulations, for each condition (i.e., set of *α*_SR+_/*α*_SR−_ and *α*_IR+_/*α*_IR−_ at the center of the square pixel), in reference to the rightmost color bar. The white cross indicates the set of *α*_+_/*α*_−_ ratios that gave the best performance (*α*_+_/*α*_−_ = 4 and 0.25 for the SR-based and IR-based systems, respectively) among those examined in this panel. Note that the minimum value of the color bar is not 0. Also, the maximum and minimum values of the color bar do not match the highest and lowest performances in this panel; rather, they were set so that the highest and lowest performances in the simulations of the same task with different parameters and/or model architecture (i.e., not only SR+IR but also SR+SR and IR+IR) shown in this panel and Figures 4 and 6 can be covered. ***B***, The black solid line, gray thin error bars, and black thick error bars, respectively, show the mean, SD (normalized by *n*; same hereafter), and SEM (approximated by SD/√*n*; same hereafter) of the performance over *n *=* *100 simulations for the conditions where *α*_+_/*α*_−_ for SR system times *α*_+_/*α*_−_ for IR system was equal to 1 (i.e., the conditions on the horizontal diagonal in ***A***). ***C***, Frequency (number of times) that each condition gave the best mean performance over 100 simulations when 100 simulations for each condition were executed 50 times (including the one shown in ***A***, ***B***).

Simulations were conducted *n *=* *100 times for the reward navigation tasks unless otherwise mentioned, or 1000 times for the two-stage tasks, for each condition by using MATLAB. For the conditions shown in Figure 3*C*, 50 sets of 100 simulations (including the one shown in Fig. 3*A*,*B*) were conducted. For the simulations where the learning rates from positive and negative TD-RPEs in the two SR-based systems or the two IR-based systems were individually varied without keeping *α_i_*_+_ + *α_i_*_−_ constant (Fig. 8), condition with (*α*_1+_, *α*_1−_, *α*_2+_, *α*_2−_) = (*x*, *y*, *z*, *w*) and condition with (*α*_1+_, *α*_1−_, *α*_2+_, *α*_2−_) = (*z*, *w*, *x*, *y*) were theoretically identical. Therefore, unless (*x*, *y*) = (*z*, *w*), 50 simulations were conducted for each condition and the resulting performance scores were merged (i.e., in total 100 simulations). Probabilities and pseudorandom numbers were implemented by “rand,” “randn,” and “randperm” functions of MATLAB. SD was calculated with normalization by *n*, and SEM was approximately calculated as SD/√*n*.

### Code accessibility

The code described in the paper is freely available online at https://github.com/kenjimoritagithub/sr5. The code is available as Extended Data 1.

10.1523/ENEURO.0422-22.2023.ed1 Extended Data 1 The code described in the present paper. The content is described in the readme.txt file. Download Extended Data 1, ZIP file.

## Results

### Performance of combined SR-based and IR-based systems, with learning rate ratio varied

We simulated a reward navigation task in a two-dimensional grid space, in which reward location changed over time (Fig. 1), and examined the performance of an agent consisting of two systems, which adopted SR or IR and may have different ratios of learning rates for positive versus negative TD-RPEs; the two systems developed system-specific state values, and their average (named the integrated state value) was used for action selection and TD-RPE calculation (Fig. 2; for details, see Materials and Methods). We first examined the case in which one system employed the SR, whereas the other adopted the IR, systematically varying the ratio of learning rates from positive and negative TD-RPEs for each system, denoted as *α*_SR+_/*α*_SR−_ for the SR-based system and *α*_IR+_/*α*_IR−_ for the IR-based system. The sums of the learning rates from positive and negative TD-RPEs (i.e., *α*_SR+_ + *α*_SR−_ and *α*_IR+_ + *α*_IR−_) were kept constant at 1, and the inverse temperature and the time discount factor were also kept constant at *β* = 5 and *γ* = 0.7. We counted how many rewards the agent obtained during the task as a measure of performance, and examined how the obtained rewards, averaged across 100 simulations for each condition, varied depending on the learning rate ratios in both systems.

**Figure 4.**
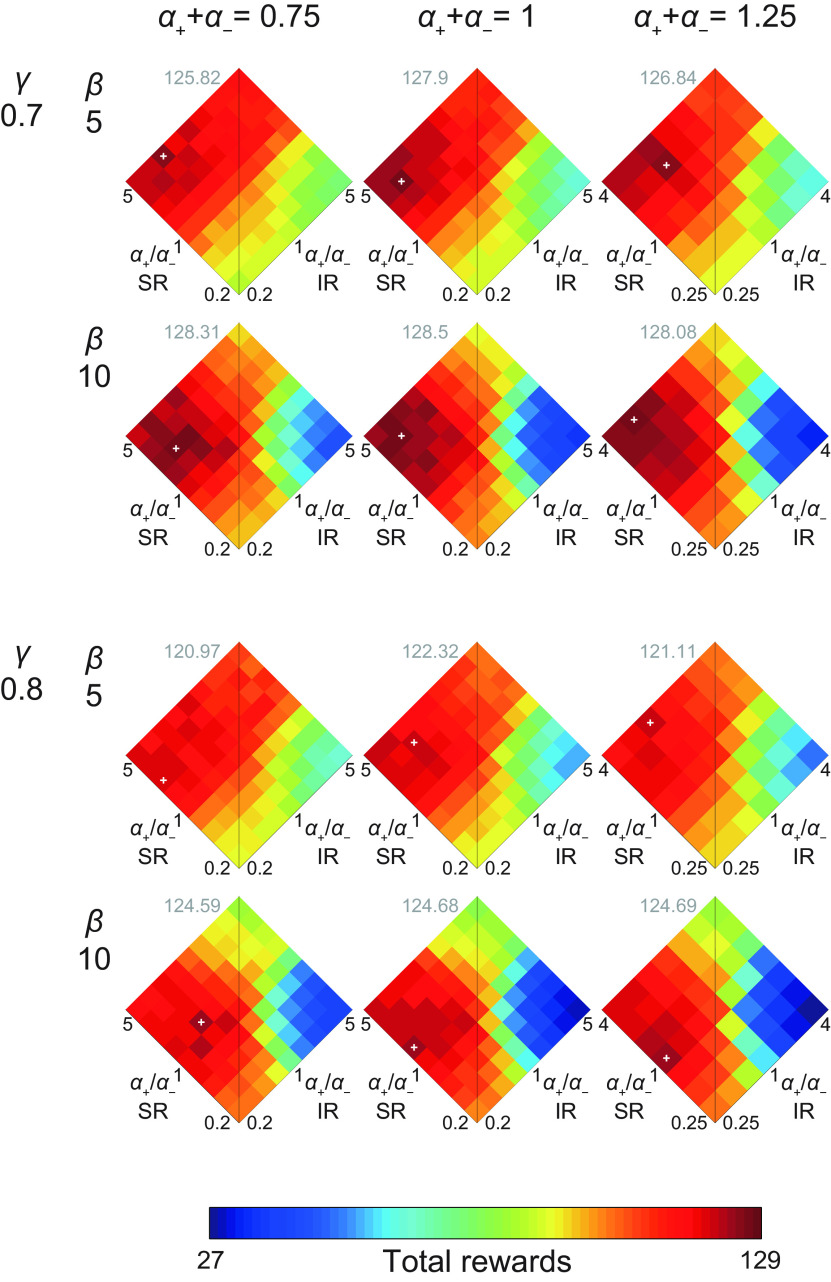
Performance (mean total rewards) of the model consisting of an SR-based system and an IR-based system with various sets of parameters. The sum of the learning rates from positive and negative TD-RPEs (*α*_+_ + *α*_−_) was varied over 0.75, 1, and 1.25. The inverse temperature *β* was varied over 5 and 10. The time discount factor *γ* was varied over 0.7 and 0.8. The ratio of positive-/negative-error-based learning rates (*α*_+_/*α*_−_) was varied over 0.2, 0.25, 1/3, 0.5, 1, 2, 3, 4, and 5 for the cases with *α*_+_ + *α*_−_ = 0.75 or 1, but 0.2 and 5 were omitted for the cases with *α*_+_ + *α*_−_ = 1.25 to avoid learning rate larger than 1. The color-performance correspondence is the same as in Figure 3*A*. The white cross in each panel indicates the set of *α*_+_/*α*_−_ ratios that gave the best performance among those examined in that condition, and the gray number near the top of each panel indicates the best performance. The panel with *α*_+_ + *α*_−_ = 1, *β* = 5, and *γ* = 0.7 shows the same results as shown in Figure 3*A*.

Figure 3*A* shows the results. As shown in the figure, the performance greatly varied depending on the conditions, and the best performance was achieved in the conditions in which *α*_SR+_/*α*_SR−_ was high (larger than 1) and *α*_IR+_/*α*_IR−_ was low (smaller than 1), i.e., the SR-based system learned mainly from positive TD-RPEs whereas the IR-based system learned mainly from negative TD-RPEs. Figure 3*B* shows the SD and SEM for the conditions where *α*_SR+_/*α*_SR−_ times *α*_IR+_/*α*_IR−_ was equal to 1 (i.e., the conditions on the horizontal diagonal in Fig. 3*A*), and Figure 3*C* shows the frequency (number of times) that each combination of *α*_SR+_/*α*_SR−_ and *α*_IR+_/*α*_IR−_ gave the best performance (mean total rewards) over 100 simulations when 100 simulations for each combination were executed 50 times (including the one shown in Fig. 3*A*,*B*). As shown in these figures, the peak of the performance was rather broad, but was mostly in the range where *α*_SR+_/*α*_SR−_ was large and *α*_IR+_/*α*_IR−_ was small. We also examined the cases where the sums of the learning rates from positive and negative TD-RPEs were increased (1.25) or decreased (0.75), and also the inverse temperature and the time discount factor were varied (*β* = 5 or 10 and *γ* = 0.7 or 0.8). As shown in Figure 4, the result that combination of the SR-based system learning mainly from positive TD-RPEs and the IR-based system learning mainly from negative TD-RPEs achieved good performance was preserved across these parameter changes.

### Learning profiles of the model with different combinations of the learning rate ratios

**Figure 5.**
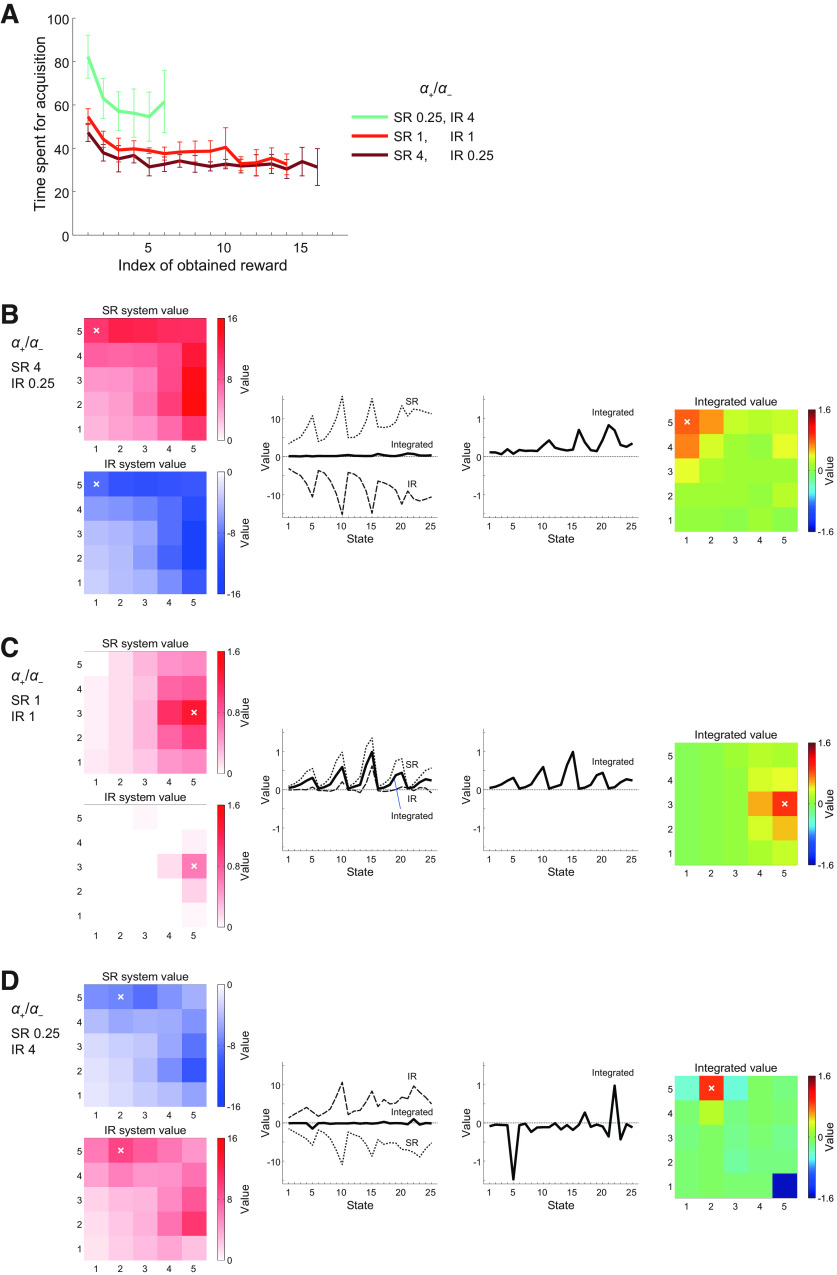
Learning profiles of the model consisting of an SR-based system and an IR-based system with different combinations of the ratios of positive-/negative-error-based learning rates in each system. Three cases included in Figure 3*A* [where the sum of the learning rates from positive and negative TD-RPEs (*α*_+_ + *α*_−_) in each system was 1, the inverse temperature *β* was 5, and the time discount factor *γ* was 0.7] were analyzed. ***A***, Mean learning curves. The curves indicate the mean time (number of time steps) used for obtaining the first, second, third… reward placed in each of the second to the ninth rewarded epoch (horizontal axis), averaged across simulations and also across the eight (from the second to the ninth) rewarded epochs. Only the cases in which reward was obtained in not smaller than a quarter of a total of 100 simulations in all of the eight epochs were plotted. The brown, red, and blue-green curves correspond to the conditions with (*α*_+_/*α*_−_ for SR-system, *α*_+_/*α*_−_ for IR-system) = (4, 0.25), (1, 1), and (0.25, 4), respectively (the colors match those in the corresponding pixels in Fig. 3*A*). The error bars indicate ±SD across the eight epochs (after taking the averages across simulations). ***B–D***, Examples of the system-specific state values and the integrated state values. Panels in ***B–D*** correspond to the conditions with (*α*_+_/*α*_−_ for SR-system, *α*_+_/*α*_−_ for IR-system) = (4, 0.25), (1, 1), and (0.25, 4), respectively. The left images show the system-specific state values (top: SR-based system, bottom: IR-based system) as a heat map on the 5 × 5 grid space, and the left graphs also show the system-specific state values (dotted line: SR-based system, dashed line: IR-based system) together with the integrated state values (thick solid line), with the horizontal axis indicating 25 states [1–5 correspond to (1,1)–(5,1) in the grid space; 6–10 correspond to (1,2)–(5,2), and so on]. Values just after the agent obtained the last reward in the last (ninth) rewarded epoch (indicated by the white crosses in the left and right panels) in single simulations, in which that reward was placed at the special candidate state in that epoch, were shown. Note the differences in the vertical scales between the left graphs in ***B*** or ***D*** and the left graph in ***C***. The right graphs show only the integrated state values, with the vertical axes in ***B*** and ***D*** enlarged as compared with the left graphs, and the right images also show the integrated state values as a heat map on the 5 × 5 grid space.

Going back to the case with original parameter values used in Figure 3 (*α*_+_ + *α*_−_ = 1, *β* = 5, and *γ* = 0.7), we examined the learning curves of the model in the case where the ratio of positive versus negative TD-RPE-based learning rate was respectively high (4) and low (0.25) in the SR-based and IR-based systems (i.e., the case achieving good performance), compared with the cases where the ratio was 1 in both systems or the ratio was low (0.25) and high (4) in the SR-based and IR-based systems. Figure 5*A* shows the mean learning curves in each case, i.e., the mean time (number of time steps) used for obtaining the first, second, third… reward placed in each rewarded epoch, averaged across eight (from the second to the ninth) rewarded epochs and also across simulations (if a reward was placed in the second epoch and obtained in the third epoch, for example, it was regarded as the last reward in the second epoch rather than the first reward in the third epoch). Only the cases in which reward was obtained in not smaller than a quarter of (25 out of total 100) simulations in all of the eight epochs were plotted, to see the general average behavior of the model. Comparing the case with high *α*_SR+_/*α*_SR−_ and low *α*_IR+_/*α*_IR−_ (brown line) and the case with low *α*_SR+_/*α*_SR−_ and high *α*_IR+_/*α*_IR−_ (blue-green line), the time consumed for obtaining the first reward in an epoch, as well as the asymptotic value of the time spent for reward acquisition, were much longer in the latter case. The case with equal ratios for both systems (red line) showed an intermediate behavior.

We also looked at how each system developed system-specific values of the states, again in the three cases where *α*_+_/*α*_−_ for the SR-based and IR-based systems were 4 and 0.25, 1 and 1, or 0.25 and 4. Specifically, we looked at single-simulation examples of those values just after the agent obtained the last reward in the last (ninth) rewarded epoch in single simulations, in which that reward was placed at the “special candidate state” (with high probability of becoming a rewarded state; see Materials and Methods) in that epoch, in the three cases. Figure 5*B* shows the good-performance case where *α*_SR+_/*α*_SR−_ was high (4) and *α*_IR+_/*α*_IR−_ was low (0.25). The left images and graphs show the system-specific values (together with the integrated values in the graphs), and the right graphs and images show the integrated values. As shown in the figure, the system-specific state values of the SR-based system (Fig. 5*B*, left graphs, dotted lines) were all positive whereas those of the IR-based system (dashed lines) were all negative, and these two look largely symmetric. This seems in line with the observation (Cui et al., 2013) that the striatal direct-pathway and indirect-pathway neurons showed concurrent activation. Meanwhile, the average of these two system-specific values, i.e., the integrated state values (Fig. 5*B* left and right graphs, thick solid lines; also shown as a heat map in Fig. 5*B*, right images), have much smaller magnitudes but show a clear pattern, which is expected to approximate the true state values under the policy that the agent was taking. Figure 5*C* shows the case where both *α*_SR+_/*α*_SR−_ and *α*_IR+_/*α*_IR−_ were 1. In this case, both systems developed similar values. Figure 5*D* shows the case where *α*_SR+_/*α*_SR−_ was low (0.25) and *α*_IR+_/*α*_IR−_ was high (4). In this case, the SR-system-specific and IR-system-specific values were negative and positive, respectively, in contrast to the case of Figure 5*B* with high *α*_SR+_/*α*_SR−_ and low *α*_IR+_/*α*_IR−_. The integrated values have comparable magnitudes to the other two cases, but were negative in many states. Comparing the right images of Figure 5*B*,*D*, the peak of the value function looks sharper in the latter, presumably reflecting the smaller degree of positive TD-RPE-dependent learning in the SR-based system.

### Cases where both systems employ only the IR or only the SR

**Figure 6.**
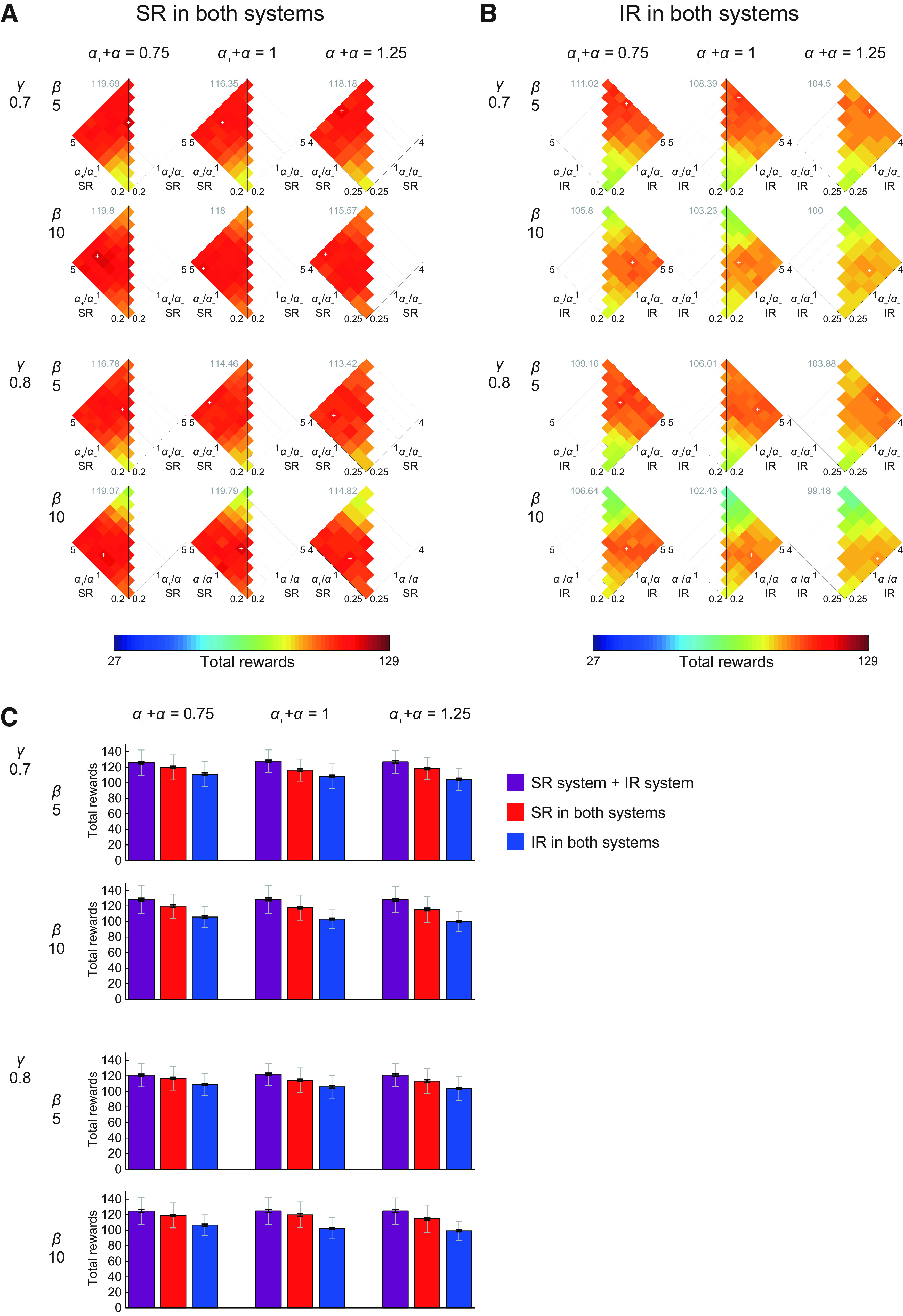
Performance of the model consisting of two systems, both of which employed the same way of state representation. Results in the case where both systems employed only the SR (***A***) or only the IR (***B***) are shown. Configurations are the same as those in Figure 4 [white cross: the best-performance set of *α*_+_/*α*_−_ ratios in each panel; gray number: the best performance (total rewards)]. If the *α*_+_/*α*_−_ ratio was equal between both systems, the two systems behaved in exactly the same manner, and thus such conditions (on the vertical lines) were equivalent to having only a single system. Also, only the left (***A***) or right (***B***) side is shown, because “*α*_1+_/*α*_1−_ = 0.2 and *α*_2+_/*α*_2−_ = 3,” for example, are equivalent to “*α*_1+_/*α*_1−_ = 3 and *α*_2+_/*α*_2−_ = 0.2” given that both systems employed the same representation. The left (***A***) and right (***B***) placement was made so as to facilitate visual comparisons with Figure 4. ***C***, Comparison of the maximum performances (mean total rewards) of the cases where one system employed the SR while the other employed the IR (purple), both systems employed the SR (red), and both systems employed the IR (blue) for each examined set of the time discount factor (*γ*), inverse temperature (*β*), and the sum of learning rates for positive and negative TD-RPEs (*α*_+_ + *α*_−_). The bar lengths indicate the mean performance over 100 simulations for the condition that gave the best mean performance, and the black thick and gray thin error bars indicate ±SEM and ±SD for that condition, respectively.

We also examined the cases where both systems employed only the SR, or only the IR. Figure 6*A*,*B* show the performance results for the SR only and IR only cases, respectively, for the same sets of parameters as shown in Figure 4 for the case of SR and IR combination. Notably, if the ratio of positive versus negative TD-RPE-based learning rates was equal between both systems that employ only the SR or IR, the two systems behaved in exactly the same manner, and thus such conditions (on the vertical lines in Fig. 6*A*,*B*) were equivalent to having only a single system. Also, Figure 6*A*,*B* only show either the left or right side, because “*α*_1+_/*α*_1−_ = 0.2 and *α*_2+_/*α*_2−_ = 3,” for example, are equivalent to “*α*_1+_/*α*_1−_ = 3 and *α*_2+_/*α*_2−_ = 0.2” given that both systems 1 and 2 employed the same representation (SR or IR) so that we examined only one of these, and Figure 6*A* shows the left side whereas Figure 6*B* shows the right side just because such arrangements might facilitate visual comparisons with Figure 4, where the ratios for the SR-based system and the IR-based system were plotted on the axes rising to the left and the right, respectively.

Comparing Figure 6*A*,*B*, SR-SR combination generally achieved better performance than IR-IR combination. This may not be surprising, given that a known feature of SR-based learning is sensitive adaptation to changes in rewards in the environments (Momennejad et al., 2017; Russek et al., 2017), which occurred in our simulated task. Besides, during the initial no-reward epoch, the SR-based system could acquire, through TD learning of SR features, an SR under the random policy, which presumably acted as beneficial “latent learning” (Dayan, 1993), while the IR-based system could not learn anything. Likewise, although the location of special reward candidate state and thus the optimal policy varied from epoch to epoch and SR is a policy-dependent representation, SR features acquired and updated in the agent should contain information about the basic structure of the grid space, which could help reward navigation throughout the task. Next, whether having two systems with different ratios of positive and negative TD-RPE-based learning rates was advantageous appears to be not clear in the cases where both systems employed only the SR or IR. Last but not least, the best combination of the SR-based and IR-based systems in the above, i.e., the combination of the SR-based system learning mainly from positive TD-RPEs and the IR-based system learning mainly from negative TD-RPEs outperformed the combination of two SR-based systems or two IR-based systems that were so far examined (compare Figs. 4 and Fig. 6*A*,*B*; also shown in Fig. 6*C*). We refer to such a combination of SR-based and IR-based systems as the combination of appetitive SR-based and aversive IR-based systems. Notably, however, in the combination that we examined, the SR-based and IR-based systems did not exclusively learn from positive and negative TD-RPEs but just mainly learned from each of them (and so also learned from the opposite TD-RPEs to a lesser extent). We did not examine combinations of exclusively appetitive and aversive systems, because targeting of the direct and indirect BG pathways by different cortical neuron types and/or regions have been suggested to be uneven but not exclusive, although (nearly) exclusive combination could exist in some parts of cortico-BG circuits or other brain circuits and/or under certain pathologic conditions.

### More comprehensive examination of the parameter space

**Figure 7.**
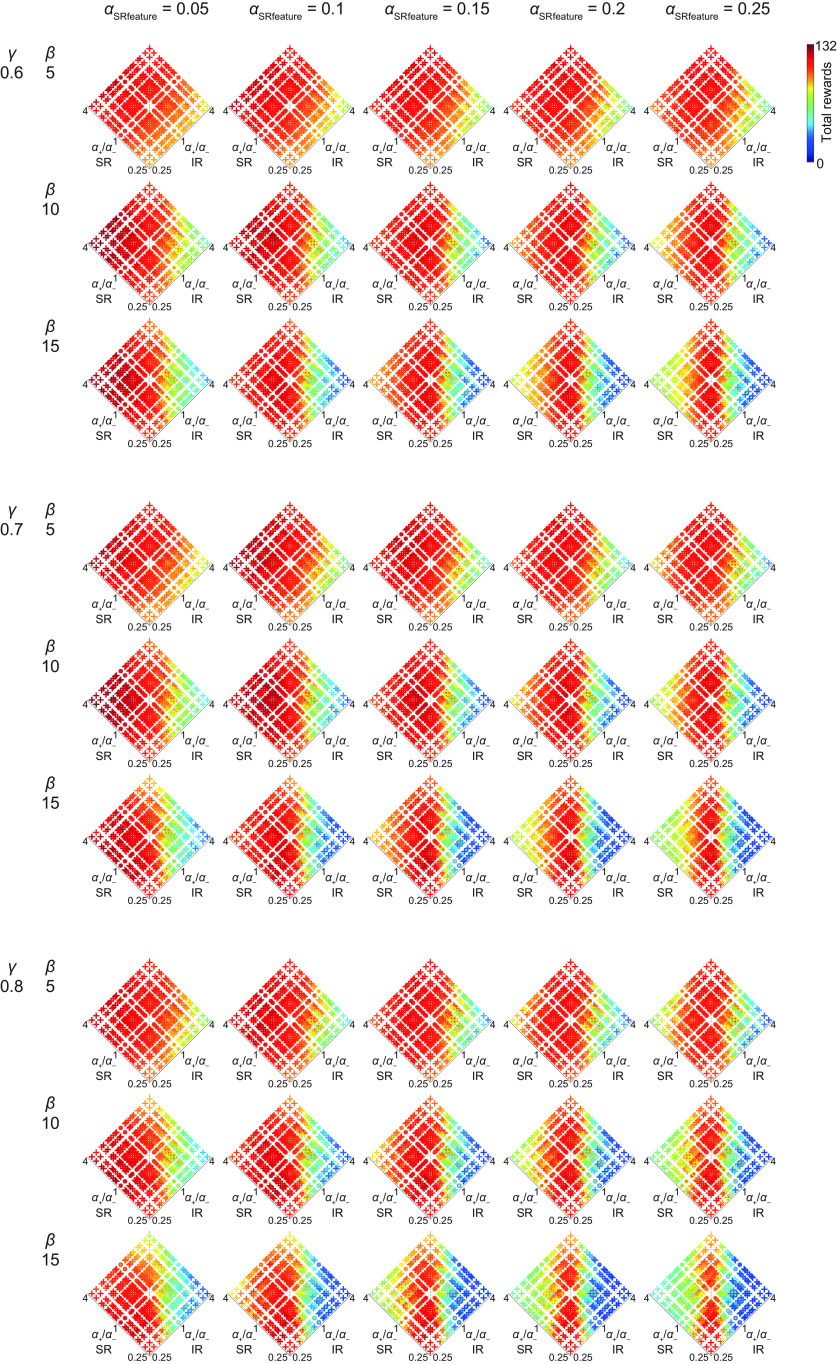
Performance of the model consisting of SR-based and IR-based systems in a broader parameter space. The learning rate from positive or negative TD-RPEs in each system was freely varied from 0.2, 0.35, 0.5, 0.65, or 0.8. Each row of panels shows the mean performance for each set of time discount factor (*γ*) and inverse temperature (*β*; shown in the left), varying the learning rate for the update of SR features (*α*_SRfeature_, shown in the top), projected onto the plane consisting of the ratios of the learning rates from positive and negative TD-RPEs in the two systems (*α*_SR+_/*α*_SR−_ and *α*_IR+_/*α*_IR−_). There were 25 cases with *α*_SR+_/*α*_SR−_ = *α*_IR+_/*α*_IR−_ = 1, which were difficult to draw on the single point (1, 1) in this projected plane and thus omitted. In the cases with *α*_SR+_/*α*_SR−_ = 1 or *α*_IR+_/*α*_IR−_ = 1 but not *α*_SR+_/*α*_SR−_ = *α*_IR+_/*α*_IR−_ = 1, there were five cases having the same set of (*α*_SR+_/*α*_SR−_, *α*_IR+_/*α*_IR−_; because *α*_+_/*α*_−_ = 1 corresponds to five cases with *α*_+_ = *α*_−_ = 0.2, 0.35, 0.5, 0.65, or 0.8), and these five cases were drawn by concentric circles with larger radius corresponding to larger learning rate. In the cases other than the special cases mentioned just above, each case was drawn by a cross. The color of the symbols (cross or circle) indicates the mean performance, in reference to the color bar in the top right. Extended Data Figure 7-1, left column, shows the sets of learning rate parameters that gave top ten mean performance for each set of time discount factor and inverse temperature.

**Figure 8.**
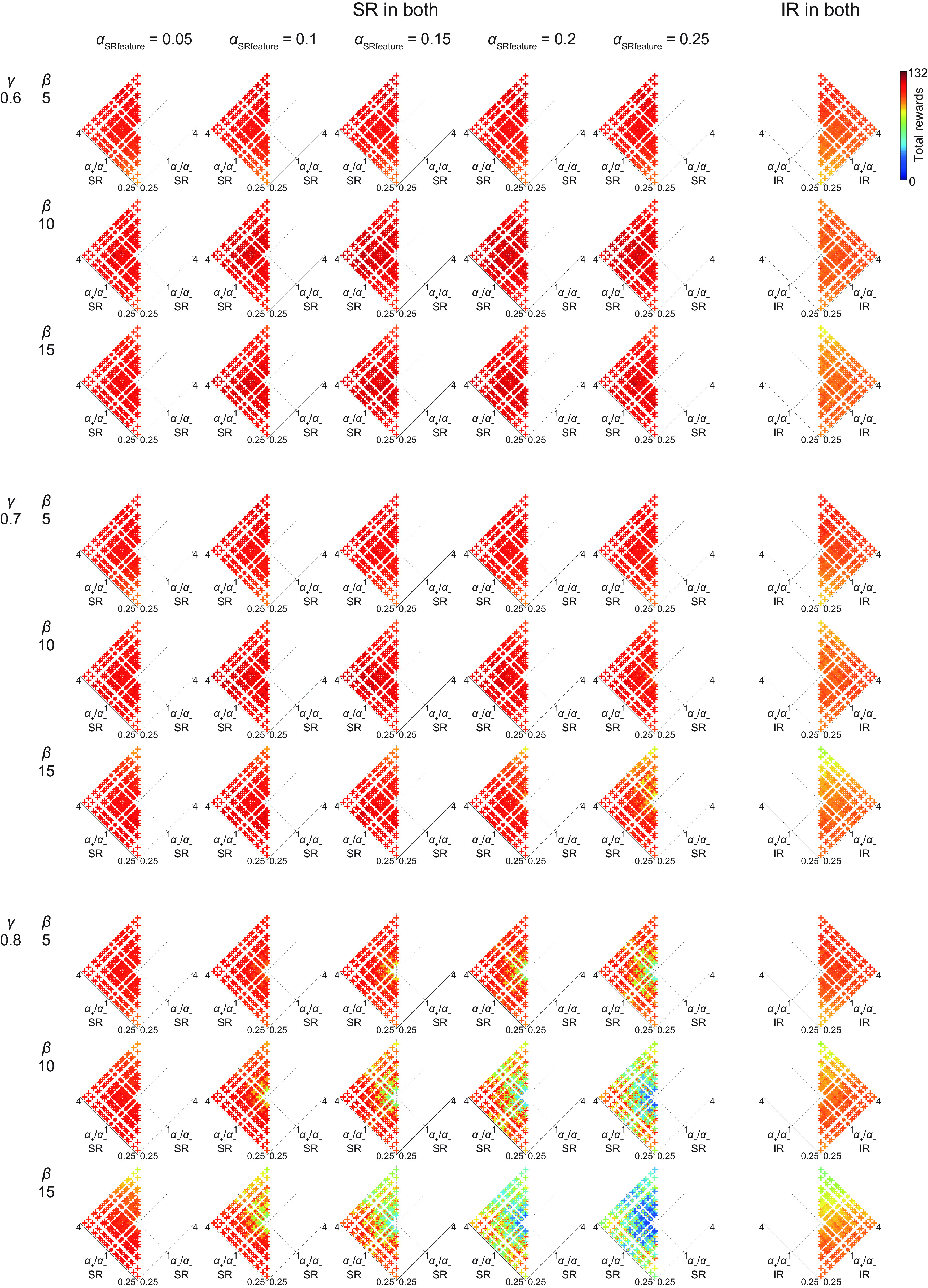
Performance of the model consisting of two SR-based systems or two IR-based systems in a broader parameter space. Each row of panels shows the mean performance for each set of time discount factor (*γ*) and inverse temperature (*β*; shown in the left), varying the learning rate for the update of SR features (*α*_SRfeature_, shown in the top) for the cases with two SR-based systems (five panels from the left), projected onto the plane consisting of the ratios of the learning rates from positive and negative TD-RPEs in the two systems (*α*_1+_/*α*_1−_ and *α*_2+_/*α*_2−_). The cases with *α*_1+_/*α*_1−_ = *α*_2+_/*α*_2−_ = 1 were not drawn, and the cases with *α*_1+_/*α*_1−_ = 1 or *α*_2+_/*α*_2−_ = 1 but not *α*_1+_/*α*_1−_ = *α*_2+_/*α*_2−_ = 1 were drawn by concentric circles, and all the other cases were drawn by crosses, in a similar manner to Figure 7. The color of the symbols (cross or circle) indicates the mean performance, in reference to the color bar in the top right. Only the left or right side is shown for the cases with two SR-based or IR-based systems, respectively, for the same reason as in Figure 6. Extended Data Figure 7-1, middle and right columns, shows the sets of learning rate parameters that gave top ten mean performance for each set of time discount factor and inverse temperature in the models consisting of two SR-based systems (middle) and two IR-based systems (right). Extended Data Figure 8-1 shows the sets of learning rates giving top ten mean performance for the cases with (*γ*, *β*) = (0.5, 15), (0.5, 20), and (0.6, 20) in the model consisting of two SR-based systems.

So far, we examined the cases where the sum of the learning rates from positive and negative TD-RPEs (*α*_+_ + *α*_−_) in both systems was fixed at one of the three values (0.75, 1, or 1.25) and also the learning rate for the update of SR features (see Materials and Methods) was fixed at 0.05. In order to examine the parameter space more widely, we varied the learning rate from positive or negative TD-RPEs in each system freely from 0.2, 0.35, 0.5, 0.65, or 0.8 and the learning rate for the update of SR features from 0.05, 0.1, 0.15, 0.2, or 0.25, and also varied the time discount factor (*γ* = 0.6, 0.7, or 0.8) and the degree of choice exploitation over exploration (inverse temperature; *β* = 5, 10, or 15). Figure 7 shows the mean performance of the model consisting of SR-based and IR-based systems. Each row of the panels shows the mean performance for each set of time discount factor and inverse temperature (shown in the left), varying the learning rate for the update of SR features (shown in the top), projected onto the plane consisting of the ratios of the learning rates from positive and negative TD-RPEs in the two systems (*α*_SR+_/*α*_SR−_ and *α*_IR+_/*α*_IR−_); there were 25 cases with *α*_SR+_/*α*_SR−_ = *α*_IR+_/*α*_IR−_ = 1, which were difficult to draw on the single point (1, 1) in this projected plane and thus omitted. Figure 8 shows the mean performance of the model consisting of two SR-based systems or two IR-based systems, projected onto the plane consisting of *α*_1+_/*α*_1−_ and *α*_2+_/*α*_2−_; 25 cases with *α*_1+_/*α*_1−_ = *α*_2+_/*α*_2−_ = 1 were omitted for the same reason as above. Extended Data Figure 7-1 shows the sets of learning rate parameters that gave top ten mean performance for each set of time discount factor and inverse temperature (shown in the left) in the model consisting of SR-based and IR-based systems (left), two SR-based systems (middle), and two IR-based systems (right).

10.1523/ENEURO.0422-22.2023.f7-1 Extended Data Figure 7-1 Performance of the models consisting of two systems in a broader parameter space. The left, middle, and right columns show the results for the model consisting of SR-based and IR-based systems, two SR-based systems, and two IR-based systems, respectively. Each subtable shows the sets of learning rate parameters that gave top ten mean performance for each set of time discount factor (*γ*) and inverse temperature (*β*; shown in the left) in each of the three models. For the model consisting of SR-based and IR-based systems, cases with a combination of appetitive (*α*_+_/*α*_−_ > 1) SR-based system and aversive (*α*_+_/*α*_−_ < 1) IR-based system are shown in bold italic; notably, even in all the other cases shown in the subtables except for a case shown in italic with asterisk in the right, the *α*_+_/*α*_−_ ratio was higher in the SR-based system than in the IR-based system. Download Figure 7-1, DOCX file.

Regarding the model consisting of SR-based and IR-based systems, under the examined conditions differing in the degrees of temporal discounting and/or choice exploitation over exploration (each row of panels in Fig. 7 and subtables in Extended Data Fig. 7-1), combination of appetitive SR-based and aversive IR-based systems (shown in bold italic in Extended Data Fig. 7-1) with relatively small learning rate for SR feature update (*α*_SRfeature_) generally achieved good performance. As *α*_SRfeature_ increased, combination of the *α*_+_/*α*_−_ ratios in the two systems that achieved good performance approached to the vertical diagonal line (i.e., similar *α*_+_/*α*_−_ ratios in both systems), but the achieved good performance itself tended to decrease when *α*_SRfeature_ exceeded 0.1 or 0.15.

Regarding the models where both systems employed only SR or IR (Fig. 8; Extended Data Fig. 7-1), the SR-only models generally outperformed the IR-only models. For the SR-only model, increase in *α*_SRfeature_ up to 0.15 or 0.2 improved the performance in many cases, but further increase appears to be not generally beneficial. As for the SR-only model, the case with (*γ*, *β*) = (0.6, 15), performed well, comparably to the case with (*γ*, *β*) = (0.7, 10). So we further examined cases with (*γ*, *β*) = (0.5, 15), (0.5, 20), and (0.6, 20). Extended Data Figure 8-1 shows the sets of learning rates giving top ten mean performance for these three cases, which look largely comparable to the cases (*γ*, *β*) = (0.6, 15) or (0.7, 10). As for the IR-only model, the cases with small inverse temperature (*β* = 5) performed well. So we further examined cases with *β* = 2.5, but performance was generally worse than the cases with *β* = 5 (best mean performance was 107.2, 107.76, and 105.12 in the cases with *γ* = 0.6, 0.7, and 0.8, respectively).

10.1523/ENEURO.0422-22.2023.f8-1 Extended Data Figure 8-1 Results of additional simulations for the model consisting of two SR-based systems. Each subtable shows the sets of learning rate parameters that gave top ten mean performance for each set of time discount factor (*γ*) and inverse temperature (*β*) shown in the left. Download Figure 8-1, DOCX file.

Comparing the three types of models using SR and IR, SR only, or IR only, combination of appetitive SR-based and aversive IR-based systems with relatively small learning rate for SR feature update generally performed well in this task.

### Dependence on task properties

**Figure 9.**
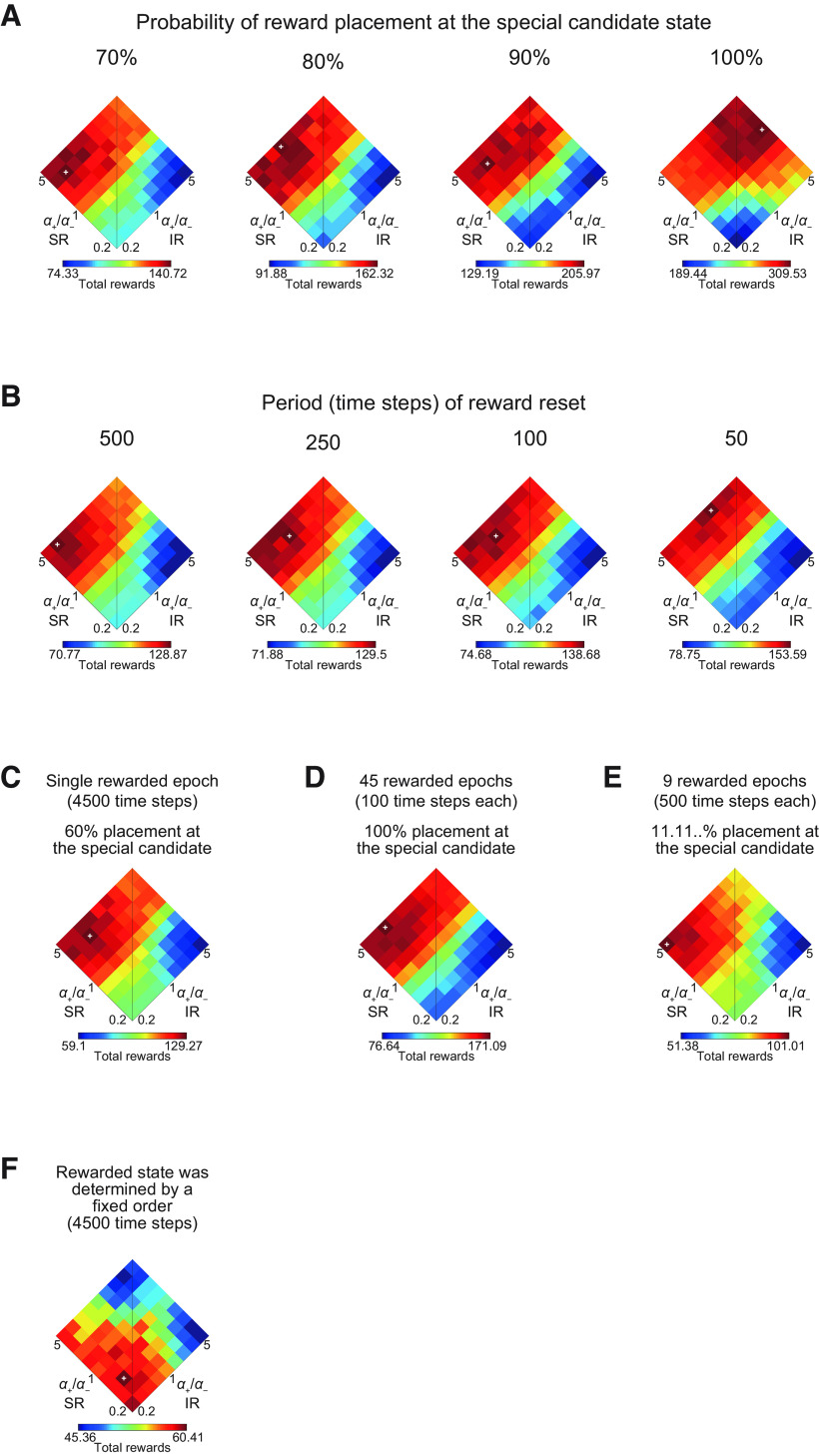
Performance of the model consisting of SR-based and IR-based system when task properties were changed. The model with the original set of parameters used in Figure 3 (*α*_SR+_ + *α*_SR−_ = *α*_IR+_ + *α*_IR−_ = 1, *β* = 5, and *γ* = 0.7) was used. The ranges of the color bars in this figure correspond to the ranges between the lowest and highest performances (i.e., the minimum and maximum mean total rewards) in the individual panels. ***A***, Performance (mean total rewards) for the cases where the probability of reward placement at the special candidate state was varied from 70% (leftmost panel), 80%, 90%, or 100% (rightmost). ***B***, Performance for the cases where reward location (state) was reset at every 500 (leftmost panel), 250, 100, or 50 (rightmost) time steps in the rewarded epochs; where reward was located was determined according to the original stochastic rule, i.e., reward was placed at the special candidate state for the epoch with 60% and placed at one of the other (normal) candidate states with 5% each. ***C***, Performance for the case where there was only a single rewarded epoch with 4500 time steps following the initial 500 time steps no-reward epoch. ***D***, Performance for the case where stochasticity of reward placement within each epoch was removed and the duration of each rewarded epoch was shortened from 500 time steps to 100 time steps while the number of rewarded epochs was increased from 9 to 45. ***E***, Performance for the case where special candidate state was abolished and reward was placed at each of the nine candidate states with equal probability (1/9 = 11.11...%). ***F***, Performance for the case where rewarded state was determined by a fixed order, namely, (5, 1), (5, 5), (1, 5), and again (5, 1), and these were repeated throughout the task.

So far, we examined the agent’s performance in the particular task. We examined how it could differ if task properties change. The original task contained a stochastic nature in a sense that reward was placed at a particular state (named the special reward candidate state) with a high probability (60%) in a given epoch but with the remaining probability reward was placed at one of the other (normal) candidate states. We examined what happened when this stochasticity was reduced or removed in the case of the model consisting of SR-based and IR-based systems with the original set of parameters used in Figures 3 and 5 (*α*_SR+_ + *α*_SR−_ = *α*_IR+_ + *α*_IR−_ = 1, *β* = 5, and *γ* = 0.7). Figure 9*A* shows the results for the cases where the probability of reward placement at the special candidate state was varied from 70% to 100%. As shown in the figure, as the stochasticity reduced, combinations of *α*_SR+_/*α*_SR−_ and *α*_IR+_/*α*_IR−_ that gave good performance gradually shifted, and when the stochasticity was totally removed, performance was good when both *α*_SR+_/*α*_SR−_ and *α*_IR+_/*α*_IR−_ were high values.

We also examined the effects of another variation in the task properties. In the original task, new reward was not placed until the agent obtained the previous reward. This property, coupled with the stochastic placement of reward, is considered to make perseveration-like behavior quite maladaptive and increase the importance of learning from negative feedbacks. We examined what happened if this property was weakened by introducing periodic resets of reward placement into the original task, again in the case of the model consisting of SR-based and IR-based systems with the original set of parameters. Figure 9*B* shows the results for the cases where reward location (state) was reset at every 500, 250, 100, or 50 time steps in the rewarded epochs; at the reset timings, reward was placed at the special candidate state for the epoch with 60% and placed at one of the other (normal) candidate states with 5% each. As shown in the figure, as the resets became more frequent, combinations of *α*_SR+_/*α*_SR−_ and *α*_IR+_/*α*_IR−_ that gave good performance again tended to gradually shift from high *α*_SR+_/*α*_SR−_ and low *α*_IR+_/*α*_IR−_ to high *α*_SR+_/*α*_SR−_ and also high *α*_IR+_/*α*_IR−_, while overall sensitivity to *α*_IR+_/*α*_IR−_ looked diminished.

We further examined three different variations of the original task. The first variation was abolishment of multiple rewarded epochs with different placements of special reward candidate state. Specifically, we examined the case where there was only a single rewarded epoch with 4500 time steps, instead of nine epochs with 500 time steps for each, following the initial 500 time-steps no-reward epoch. The special candidate state was varied only across simulations. The second variation was removal of the stochasticity of reward placement within each epoch, together with shortening of the duration of each rewarded epoch (from 500 time steps to 100 time steps) and increasing the number of rewarded epochs (from 9 to 45). The third variation was abolishment of special reward candidate state. Specifically, we came back to the original nine 500 time-steps rewarded epochs, but the probability of reward placement at the special candidate state was changed from the original 60% to 1/9 (11.11...%) so that reward was placed at each of the nine candidate states with equal probability (1/9). Figure 9*C–E* shows the performance of the model consisting of SR-based and IR-based systems with the original set of parameters in these three variations. As shown in the figures, the patterns look similar to the case of the original task (Fig. 3).

It was rather surprising that similar pattern appeared even in the last case, because reward was placed at any candidate states with equal probabilities and so it was no longer a task where the agent could learn where the special candidate state was in each epoch. However, the nine candidate states were neighboring with each other on the two edges of the grid space, and thus it would still have been the case that next reward was placed at a state near from the previously rewarded state with a relatively high probability and the agent could exploit it through positive TD-RPE-based learning. As a control, we examined a task in which positive TD-RPE-based learning was expected to have little meaning. Specifically, after 500 time-steps no-reward epoch, rewarded state was determined by a fixed order, namely, (5, 1), (5, 5), (1, 5), and again (5, 1), and these were repeated throughout the task. Figure 9*F* shows the performance of the model consisting of SR-based and IR-based systems with the original parameters. As shown in the figure, good performance, though rather low as expected, was achieved when both *α*_SR+_/*α*_SR−_ and *α*_IR+_/*α*_IR−_ were low values. This is reasonable because only quick erasing of the memory of previous reward is considered to be adaptive in this control task.

These results together indicate that the combination of appetitive SR and aversive IR systems does not always achieve good performance, but does so in certain environments where reward placement is dynamically changing but still learnable using both positive and negative TD-RPEs.

### Performance of the two-system models in the two-stage tasks

**Figure 10.**
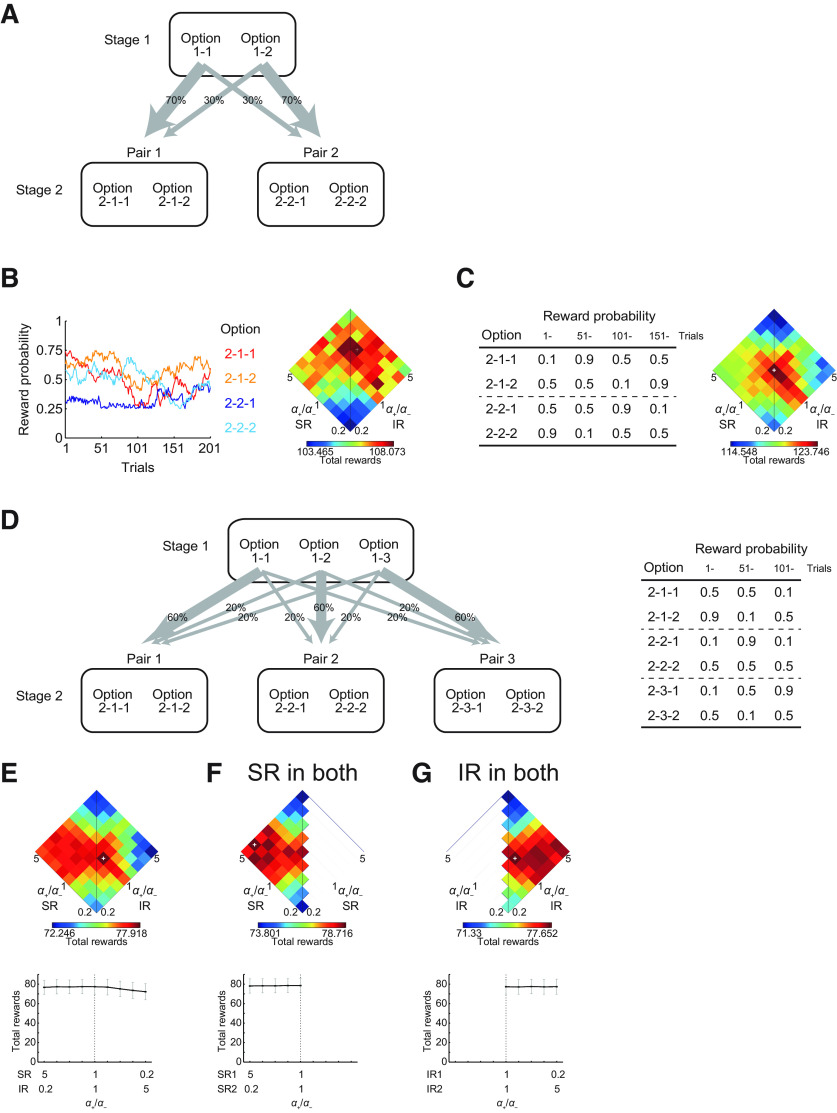
Performance of the two-system models in the two-stage tasks. ***A***, Schematic diagram of the two-stage task. Selection of one of the two first-stage options lead to one of the two pairs of second-stage options with fixed probabilities. ***B***, Left, Reward probabilities for the four second-stage options in the original two-stage task. The probability for each option was independently set according to Gaussian random walk with reflecting boundaries at 0.25 and 0.75. An example is shown. Right, Mean performance (mean total rewards over *n *=* *1000 simulations) of the model consisting of an SR-based system and an IR-based system, with the ratio of positive-/negative-error-based learning rates (*α*_+_/*α*_−_) for each system was varied under *α*_+_ + *α*_−_ = 1, *β* = 5, and *γ* = 1. ***C***, Left, Reward probabilities for the four second-stage options in a variant of the task. The probabilities for the four options were set to specific values, which changed three times in the task. Right, Mean performance of the model. ***D***, Left, Schematic diagram of a variant of the two-stage task, in which there were three first-stage options and three pairs of second-stage options. Right, Reward probabilities for the six second-stage options, which were set to specific values and changed two times in the task. ***E–G***, Top panels, Mean performance of the model consisting of SR-based and IR-based systems (***E***), two SR-based systems (***F***), or two IR-based systems (***G***), in the task variant with three first-stage options and three pairs of second-stage options. Bottom graphs, Mean (black solid line), SEM (black thick error bars; though hardly visible), and SD (gray thin error bars) of the performance over *n *=* *1000 simulations for the conditions where *α*_+_/*α*_−_ for SR system times *α*_+_/*α*_−_ for IR system was equal to 1 (i.e., the conditions on the horizontal diagonal in the top panels).

Whether humans or animals take model-based/goal-directed or model-free behavior has been widely examined by using the so-called two-stage (or two-step) tasks (Daw et al., 2011; Groman et al., 2019a). Therefore, we also examined how our models consisting of two systems performed in such tasks. We simulated the two-stage task (Daw et al., 2011). At the first stage, there were two choice options. Selection of each of these two options lead to one of two pairs of second-stage options with fixed probabilities (70% or 30%; Fig. 10*A*). Then, selection of one of the second-stage options lead to reward or no-reward outcome. The probability of reward for each second-stage option was independently set according to Gaussian random walk with reflecting boundaries at 0.25 and 0.75 (an example is shown in Fig. 10*B*, left). The IR-based system learned the value of each first-stage and second-stage option through RPE-based updates. The SR-based system had the SR of the first-stage and second-stage options, and leaned their values through RPE-based updates of the weights of the approximate value function. The initial values of the SR matrix were set incorporating the probabilities of the transitions from the first stage to the second stage and also assuming random choice policy at the second stage, and the SR matrix was updated by using the prediction error of SR features.

Figure 10*B*, right panel, shows the performance of the model with (*α*_+_ + *α*_−_ = 1, *β* = 5, and *γ* = 1) and the ratio of the learning rates for positive and negative RPEs in each system (*α*_SR+_/*α*_SR−_ and *α*_IR+_/*α*_IR−_) varied. As shown in the figure, combination of appetitive SR and aversive IR systems did not give a good performance in this task. We also examined a variant of the task, where the probabilities of reward for the second-stage options were set to specific values, which changed three times during the task (Fig. 10*C*, left), so that differences among the values and their temporal changes could be clearer than the original case using random walk. Figure 10*C*, right panel, shows the performance of the same model, and here again combination of appetitive SR and aversive IR systems did not give a good performance.

A possible reason why the good performance of such a combination observed in the original navigation task was not generalized is that the two-stage tasks imposed only selection from two options at both stages while the navigation task imposed selection from more than two options. In the case with only two choice options, an increase in the choice probability of one option simultaneously means the same magnitude of decrease in the choice probability of another option. Intuitively, generalization of learning from positive feedbacks for one option appears to have limited merit if choice probabilities are determined by the value difference between two options and there is no other option to which generalization can be applied. Therefore, we simulated a variant of two-stage task, in which there were three, rather than two, first-stage options and three pairs of second-stage options (Fig. 10*D*). Figure 10*E* shows the performance of the same model in this task. As shown in the figure, in this case, combination of appetitive SR-based system and aversive IR-based system achieved a relatively good performance. Conditions with similar intermediate *α*_+_/*α*_−_ ratios for both systems, or even with higher *α*_+_/*α*_−_ ratio for the IR system than for the SR system, achieved a good, or even better, mean performance, but shifts from such conditions toward appetitive IR-based and aversive SR-based systems resulted in a rapid decrease in the performance while shifts toward appetitive SR-based and aversive IR-based systems resulted in a milder decrease in the performance, although the overall range of the changes in the mean performance was rather small. Given this, we considered that the good performance of the combination of appetitive SR-based and aversive IR-based systems in the original navigation task could be generalized, to a certain extent, to this version of two-stage task with more than two options.

Figure 10*F*,*G* shows the performance of the models in which both of the two systems employed only SR or IR, respectively. The ranges of the performance of these two types of models look comparable with each other, and also comparable to the performance range of the model consisting of SR-based and IR-based systems. Therefore, the superiority of the solely SR-based model over the solely IR-based model, and also the superiority of the combined SR-based and IR-based systems over the solely SR-based (or IR-based) system, observed in the original navigation task was not generalized to this two-stage task. It has been pointed out (Kool et al., 2016) that model-based control actually provides little performance benefits in the original two-stage task (Daw et al., 2011) as well as in several variants. Although model-based control can still be beneficial in certain two-stage tasks (Kool et al., 2016), advantages of model-based or SR-based control, and potentially also of the combined SR-based and IR-based systems, might appear more clearly in tasks with more than two stages such as the navigation task.

## Discussion

We found that the combination of SR-based system learning mainly from positive TD-RPEs and IR-based system learning mainly from negative TD-RPEs showed superior performance in certain dynamic reward environments. Below we discuss possible reasons for the superiority of such a combination, and also discuss the possibility that the same combination could perform badly in certain other environments. We then show how the combination of appetitive SR-based and aversive IR-based systems seems in line with diverse anatomic and physiological findings about the cortico-BG circuits and thereby potentially explains their functional significance and underlying mechanism. We also discuss limitations of the present study, with future perspectives.

### Reasons for the superior performance of the combination of appetitive SR-based and aversive IR-based systems

As possible reasons why the combination of appetitive SR-based and aversive IR-based systems performed well in the reward navigation task, the following two are considered. The first possible reason comes from an asymmetry between positive-error-based and negative-error-based learning. When the agent unexpectedly encountered a reward at a certain state, next reward was likely to be placed at the same or nearby states with high probabilities given the structure of the task. Then, quickly revising up the value of not only the rewarded state itself but also any states from which the rewarded state could be easily reached (i.e., states having high SR feature values) would be beneficial. In other words, SR-dependent generalization of positive TD-RPE-based value updates would be beneficial. The result that the combination of appetitive SR-based and aversive IR-based systems performed relatively well in the two-stage task with three first-stage options but not in the task with two options seems in line with this, because generalization would have a larger merit in the former task variant. Next, assume that the agent obtained rewards several times at the same or nearby states (in the navigation task), and then reward was placed at a distant state. This time, the agent was likely to unexpectedly encounter reward omission. Critically, through the repetitive acquisition of rewards at the nearby states, the agent’s policy was likely to be sharpened and therefore revising down the values of only the states right on the sharpened policy would already be effective. In other words, as for negative TD-RPE-based value updates, IR-based narrow effect would be largely sufficient, and too much SR-dependent generalization could rather be harmful.

The second possible reason for the good performance of the combination of appetitive SR-based and aversive IR-based systems comes from the policy-dependence of SR, which would also be related to the last point of the above. SR reflects state transitions under the policy that has been used. Thus, when reward is relocated at a distant place and thereby optimal policy is drastically changed, SR under the policy optimized for the previous reward should significantly differ from the one under the new optimal policy, and SR becomes updated as the agent changes its policy. In fact, through TD-RPE-based learning, in contrast to direct calculation of state values by multiplication of SR and reward at each state, value function after reward relocation can in principle be approximated even with SR under the previously near-optimal policy, as well as with arbitrary state representation, as suggested previously (Russek et al., 2017). Nonetheless, SR under the previously near-optimal policy may not have fine information about state transitions near the new reward location, implying potential difficulty in learning from negative TD-RPEs for SR-based system, which could contribute to the superiority of the combination of appetitive SR-based and aversive IR-based systems, or more precisely, the inferiority of the opposite combination of aversive SR-based and appetitive IR-based systems. Looking at Figures 3*B* and 5*A*, the difference in the performance between the aversive SR and appetitive IR combination and the neutral SR and IR combination was larger than the difference between the neutral SR and IR combination and the appetitive SR and aversive IR combination. This may imply that the demerit of SR-based learning from negative TD-RPEs had a larger impact than the merit of SR-based generalization from positive TD-RPEs in this task, possibly because there were only a small number of states/actions for which generalization could be applied.

Difficulty in the case with drastic change in the goal and the optimal policy has previously been shown (Lehnert et al., 2017; see also Lehnert and Littman, 2020) for learning using successor features, which are generalization of SR (Barreto et al., 2016). Increasing the learning rate for SR feature update (*α*_SRfeature_) could potentially mitigate this issue. In our simulations with *α*_SRfeature_ varied (Fig. 7), as *α*_SRfeature_ increased, combination of the *α*_+_/*α*_−_ ratios in the two systems that achieved good performance approached to the ones with similar ratios in both systems, potentially in line with this. At the same time, however, increase in *α*_SRfeature_ did not generally improve the highest achievable performance of the model with SR-based and IR-based systems; increase up to 0.1 improved it in some but not other conditions, and further increase could rather worsen it presumably because state representation became too unstable, while increase in *α*_SRfeature_ up to 0.15 or 2 was beneficial in many cases for the model consisting of two SR-based systems (Fig. 8). Given the result that relatively small *α*_SRfeature_ was good for the model with appetitive SR-based and aversive IR-based systems, we speculate that policy-independent state representation based on the transition structure, specifically, the default representation (Piray and Daw, 2021), could alternatively be used.

While the combination of appetitive SR-based and aversive IR-based systems could perform well in certain dynamic environments presumably because of the abovementioned reasons, such a combination deviates from normative RL algorithms and could thus perform badly in certain other environments, perhaps in particular stable ones. Indeed, recent work (Sato et al., 2022) has shown that agent having appetitive SR-based and aversive IR-based systems could potentially develop obsession-compulsion cycle in a particular (stable) environment considered in (Sakai et al., 2022), and discussed that the superiority of such a combination in certain dynamic environments, shown in the present study, could potentially explain the human’s proneness to obsessive-compulsive disorder, which was suggested in (Sakai et al., 2022) based on the data of patients and healthy controls. It still remains to be clarified how the combination of appetitive SR-based and aversive IR-based systems performs well or badly under specific conditions in larger task spaces.

### Explanation for the significance and mechanism of diverse findings about the cortico-BG circuits

The superiority of the combination of SR-based appetitive and IR-based aversive learners in certain dynamic reward environments shown in our model provides a novel coherent explanation for the functional significance and underlying mechanism of diverse findings about the cortico-BG circuits, which could not be explained by previous dual-systems BG models that did not consider the diversity of the neocortex and different representations potentially used therein.

**Figure 11.**
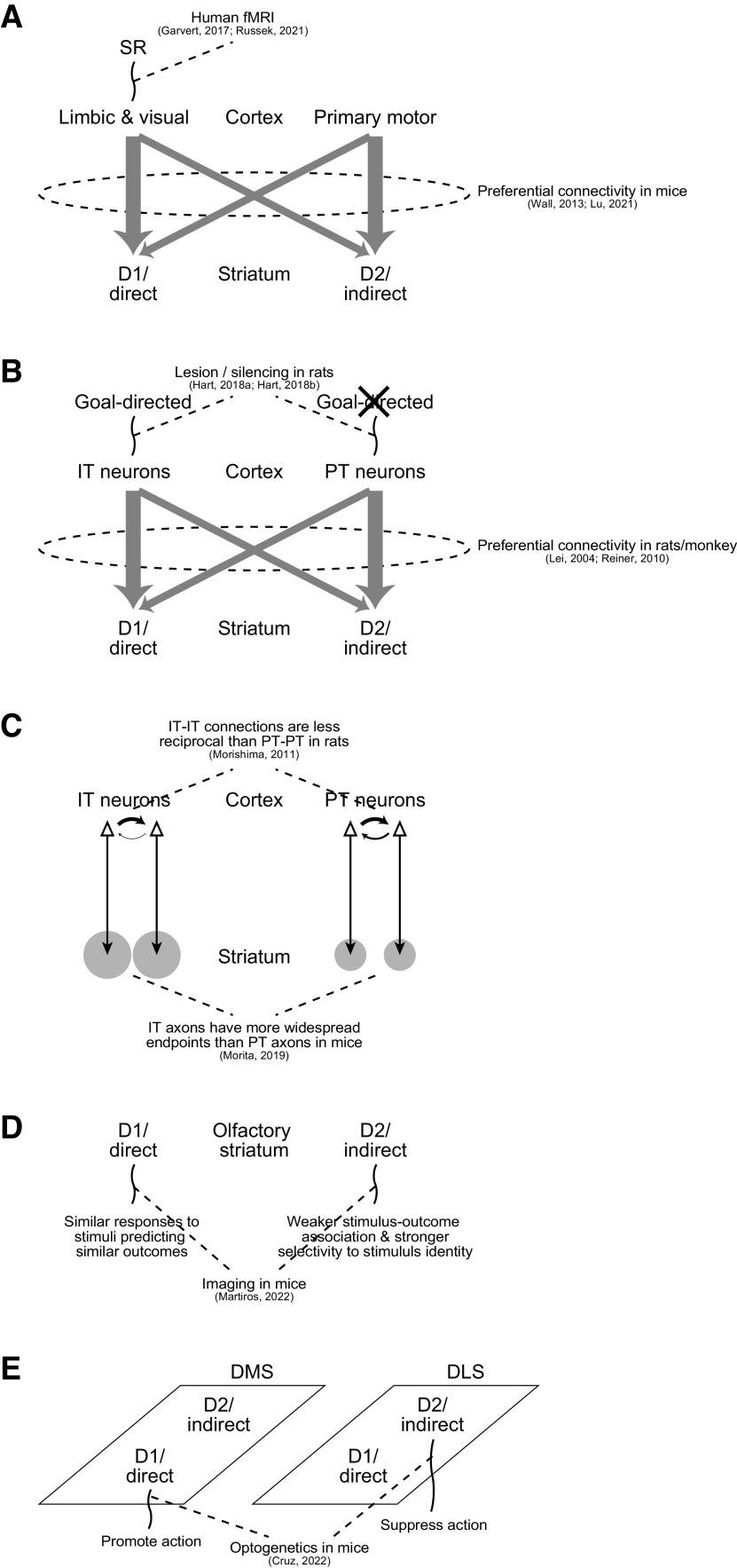
Explanation for the significance and mechanism of diverse findings about the cortico-BG circuits. ***A***, Experimentally suggested limbic/visual cortical encoding of SR and limbic/visual->D1/direct and primary motor->D2/indirect preferential connections indicate preferential use of SR in the appetitive D1/direct pathway, whose functional merit was explained by the results of our simulations. ***B***, Experimentally suggested involvement of IT-type, but not PT-type, corticostriatal neurons in goal-directed behavior and IT->D1/direct and PT->D2/indirect preferential connections also indicate preferential use of SR in the appetitive D1/direct pathway. ***C***, Less reciprocal IT-IT connections and wider IT->striatum axonal endpoints are in line with engagement of IT (rather than PT) neurons in SR-like representation. ***D***, D1 neurons’ similar responses to stimuli predicting similar outcomes and D2 neurons’ weaker stimulus-outcome association and stronger selectivity to stimulus identity in the ventral striatal olfactory tubercle could be explained by preferential use of SR-like and IR-like representations in the D1 and D2 pathways, respectively. ***E***, Engagements of D1/direct neurons in the dorsomedial striatum (DMS), a suggested locus of model-based behavior, and D2/indirect neurons in the dorsolateral striatum (DLS), a suggested locus of model-free behavior, in promotion and suppression of action, respectively, are potentially in line with the combination of appetitive SR-based and aversive IR-based systems in our model.

First, monosynaptic rabies virus tracing in mice revealed preferential connections from the limbic/visual cortices and primary motor cortex to the D1/direct and D2/indirect pathways, respectively (Wall et al., 2013; Lu et al., 2021), while human fMRI experiments found activations indicative of SR in the limbic/visual cortices, or more specifically, hippocampal–entorhinal cortex (Garvert et al., 2017) and visual cortex (Russek et al., 2021; Fig. 11*A*). These findings together indicate preferential use of SR in the D1/direct pathway (and not in the D2/indirect pathway), and the results of our simulations explain a functional merit of such a combination.

Second, electron microscopic analyses focusing on synapse sizes in rats (Lei et al., 2004) and monkey (Reiner et al., 2010) indicated preferential connections from the intratelencephalic (IT)-type and pyramidal-tract (PT)-type corticostriatal neurons to the D1/direct and D2/indirect pathways, respectively, while lesion and silencing experiments in rats (Hart et al., 2018a,b) demonstrated that prelimbic IT neurons, but not PT neurons, mediated sensitivity to outcome devaluation (Fig. 11*B*), which is a defining feature of goal-directed behavior and could potentially be achieved through SR-based learning. These findings together can again be in line with preferential use of SR in the D1/direct pathway. Notably, electrophysiological and optogenetic studies (Ballion et al., 2008; Kress et al., 2013) did not find evidence for preferential IT->D1/direct and PT->D2/indirect pathway activations, whereas a study (Morita, 2014) conducting model fitting of reported paired-pulse-ratio data (Ding et al., 2008) with certain assumptions argued that preferential activation could still potentially occur if short-term synaptic plasticity is taken into account.

Third, local connections between IT neurons were shown to be less reciprocal (i.e., more unidirectional) than those between PT neurons in rat frontal cortex (Morishima et al., 2011), and analysis of the MouseLight database (Winnubst et al., 2019) revealed that axonal projections of individual IT neurons to the striatum have on average more widespread endpoints than those of PT neurons (Morita et al., 2019; Fig. 11*C*). These properties are also in line with the possibility that IT neurons (rather than PT neurons) are engaged in SR-like representation, because SR is based on the directional transition relationships between states and also calculation of state value using SR requires access to not only that state but also all the states which are reached from that state through transitions.

Fourth, recent work examining the ventral striatal olfactory tubercle of mice (Martiros et al., 2022) found that D1 neurons showed similar responses to stimuli predicting similar outcomes whereas D2 neurons showed weaker stimulus-outcome association and stronger selectivity to stimulus identity (Fig. 11*D*). These differential responses could be explained by preferential use of SR-like and IR-like representations in the D1 and D2 pathways, respectively, in line with the superior combination in our simulations, although the olfactory tubercle is known to receive direct inputs from the olfactory bulb (Igarashi et al., 2012) and it seems unclear whether there exist region/neuron-type-dependent and pathway-dependent connection preferences homologous to those suggested for other (more dorsal) striatal region.

Last but not least, another recent study using mice (Cruz et al., 2022) found that the D2/indirect-pathway SPNs in the dorsolateral striatum (DLS), a suggested locus of habitual or model-free behavior (Yin et al., 2004; Dolan and Dayan, 2013), crucially engaged in suppression of action whereas the D1/direct-pathway SPNs in the dorsomedial striatum (DMS), a suggested locus of goal-directed/model-based behavior (Yin et al., 2005a, b; Dolan and Dayan, 2013), promote action. The combination of appetitive SR-based and aversive IR-based systems in our model appears to match this observed combination of promotive DMS and suppressive DLS (Fig. 11*E*), explaining its possible generation mechanism and functional merit.

### Limitations and perspectives

There are a number of limitations in the present work. Sets of parameters that we examined were still limited. Moreover, whether the superiority of the combination of appetitive SR-based and aversive IR-based systems observed in our navigation task is applied also to other tasks in larger task spaces, including more complex tasks, remains to be seen as mentioned above. Neurobiologically, while we considered appetitive and aversive learning in the two BG pathways, there are studies suggesting that the roles of these pathways differ not merely in the valence (Nonomura et al., 2018; Iino et al., 2020; Matamales et al., 2020; Peak et al., 2020). Also, mechanisms of TD-RPE calculation in DA neurons (Watabe-Uchida et al., 2017; Morita and Kawaguchi, 2019), as well as richer temporal dynamics and functional roles of DA suggested by recent studies (Kato and Morita, 2016; Bogacz, 2020; Hamid et al., 2021; Mikhael et al., 2022), remain to be incorporated.

A central prediction of the present work is that humans and other animals take a learning strategy that combines appetitive SR-based/model-based learning and aversive IR-based/model-free learning in situations having similarities to the reward navigation task examined here. This can in principle be tested by examining whether the behavior exhibits features of SR-based learning (cf. Momennejad et al., 2017) differently between the cases with positive and negative feedbacks, potentially also with neuroimaging (cf. Garvert et al., 2017; Russek et al., 2021).

## Synthesis

Reviewing Editor: Mark Laubach, American University Decisions are customarily a result of the Reviewing Editor and the peer reviewers coming together and discussing their recommendations until a consensus is reached. When revisions are invited, a fact-based synthesis statement explaining their decision and outlining what is needed to prepare a revision will be listed below. The following reviewer(s) agreed to reveal their identity: Stephanie Groman.

Your manuscript was reviewed by two experts, and your revisions to address the reviews from JN were considered. One reviewer felt that the revisions were adequate but did raise one concern that I think you should address. The other reviewer raised several concerns about the readability and thus potential impact of your study, and I would like to ask that you consider addressing all points that were raised if you decide to submit a revision to eNeuro.

Reviewer 1 wrote “It remains unclear how or if the current findings apply to larger task spaces or other designs.” Please add text to the Discussion section to address this concern.

Reviewer 2’s review is given below in full. Please revise the paper extensively to address all points that were raised.

Thank you for sending your paper to eNeuro.

Reviewer 2

The paper as written is almost impenetrable to a non-expert. I consider myself familiar with these approaches and the theoretical basis of the manuscript and still found it incredibly difficult to understand the results and conclusions. I believe the results are incredibly important and very interesting - particularly for the field of computational psychiatry - and would love to see this paper get the attention it deserves. I am afraid, however, that as written the manuscript will not get the credit I believe it deserves. I suspect that most of my comments could be addressed by clarifying aspects of experimental design, dependent measures and results, providing justifications for specific approaches and conclusions, and considering streamlining the manuscript.

1. The dependent measures (outside of total rewards earned) are not adequately described. For example, the y-axis on Figure 5A is “time spent for acquisition” but what this measure is never described. This is present throughout the manuscript and made it very difficult to evaluate the data being presented and the authors conclusions.

2. It would be useful if the authors stated why the reward probability for the “special” versus “normal” candidate states was needed in the Methods and Materials.

3. Figure 5A - I was surprised that the time spent for reward acquisition for the case with equal ratios for both systems was not more different from the case of high ASR+/ASR- and low AIR+/AIR-. Were the authors also surprised? Any insights into why this is the case?

4. The authors argue (Pg 12) that the combination of SR from positive TD-PEs and IR from negative TD-PEs outperformed any model and point the readers to Figure 4 and 6. However, I don’t understand how the data presented in these figures supports this conclusion. My assumption was that the alpha+/alpha- values in Figure 6A and 6B were ratios of the learning rates and that both the IR and SR are learning from both positive PEs and negative PEs. I think to support the authors claims, they would need to simulate data with a model that only uses SR-based positive PEs and IR-based negative PEs.

5. The experimental manipulations presented in Figures 7, 8, and 9 (which are never explained or justified) yield really interesting results - especially when p(reward placement) goes from stochastic to deterministic. However, I suspect most readers will gloss over these findings because the authors almost present these manipulations as control experiments or conditions.

6. I wonder if the authors might consider removing the model of the cortico-basal ganglia from the manuscript. I think this would help streamline the manuscript and improve readability.

## References

[B1] Balleine BW, O’Doherty JP (2010) Human and rodent homologies in action control: corticostriatal determinants of goal-directed and habitual action. Neuropsychopharmacology 35:48–69. 10.1038/npp.2009.131 19776734 PMC3055420

[B2] Ballion B, Mallet N, Bézard E, Lanciego JL, Gonon F (2008) Intratelencephalic corticostriatal neurons equally excite striatonigral and striatopallidal neurons and their discharge activity is selectively reduced in experimental parkinsonism. Eur J Neurosci 27:2313–2321. 10.1111/j.1460-9568.2008.06192.x 18445222

[B3] Barreto A, Dabney W, Munos R, Hunt JJ, Schaul T, van Hasselt H, Silver D (2016) Successor features for transfer in reinforcement learning. arXiv:1606.05312. 10.48550/arXiv.1606.05312.

[B4] Bogacz R (2020) Dopamine role in learning and action inference. Elife 9:e53262. 10.7554/eLife.53262 32633715 PMC7392608

[B5] Chen Y, Monaco S, Byrne P, Yan X, Henriques DY, Crawford JD (2014) Allocentric versus egocentric representation of remembered reach targets in human cortex. J Neurosci 34:12515–12526. 10.1523/JNEUROSCI.1445-14.2014 25209289 PMC6615499

[B6] Collins AG, Frank MJ (2014) Opponent actor learning (OpAL): modeling interactive effects of striatal dopamine on reinforcement learning and choice incentive. Psychol Rev 121:337–366. 10.1037/a0037015 25090423

[B7] Cruz BF, Guiomar G, Soares S, Motiwala A, Machens CK, Paton JJ (2022) Action suppression reveals opponent parallel control via striatal circuits. Nature 607:521–526. 10.1038/s41586-022-04894-9 35794480

[B8] Cui G, Jun SB, Jin X, Pham MD, Vogel SS, Lovinger DM, Costa RM (2013) Concurrent activation of striatal direct and indirect pathways during action initiation. Nature 494:238–242. 10.1038/nature11846 23354054 PMC4039389

[B9] Daw ND, Gershman SJ, Seymour B, Dayan P, Dolan RJ (2011) Model-based influences on humans’ choices and striatal prediction errors. Neuron 69:1204–1215. 10.1016/j.neuron.2011.02.027 21435563 PMC3077926

[B10] Dayan P (1993) Improving generalization for temporal difference learning: the successor representation. Neural Comput 5:613–624. 10.1162/neco.1993.5.4.613

[B11] Ding J, Peterson JD, Surmeier DJ (2008) Corticostriatal and thalamostriatal synapses have distinctive properties. J Neurosci 28:6483–6492. 10.1523/JNEUROSCI.0435-08.2008 18562619 PMC3461269

[B12] Dolan RJ, Dayan P (2013) Goals and habits in the brain. Neuron 80:312–325. 10.1016/j.neuron.2013.09.007 24139036 PMC3807793

[B13] Frank MJ, Seeberger LC, O’reilly RC (2004) By carrot or by stick: cognitive reinforcement learning in parkinsonism. Science 306:1940–1943. 10.1126/science.1102941 15528409

[B14] Gardner MPH, Schoenbaum G, Gershman SJ (2018) Rethinking dopamine as generalized prediction error. Proc R Soc B 285:20181645. 10.1098/rspb.2018.1645 PMC6253385 30464063

[B15] Garvert MM, Dolan RJ, Behrens TE (2017) A map of abstract relational knowledge in the human hippocampal-entorhinal cortex. Elife 6:e17086. 10.7554/eLife.17086 28448253 PMC5407855

[B16] Gershman SJ, Moore CD, Todd MT, Norman KA, Sederberg PB (2012) The successor representation and temporal context. Neural Comput 24:1553–1568. 10.1162/NECO_a_00282 22364500

[B17] Groman SM, Massi B, Mathias SR, Curry DW, Lee D, Taylor JR (2019a) Neurochemical and behavioral dissections of decision-making in a rodent multistage task. J Neurosci 39:295–306. 10.1523/JNEUROSCI.2219-18.2018 30413646 PMC6325257

[B18] Groman SM, Keistler C, Keip AJ, Hammarlund E, DiLeone RJ, Pittenger C, Lee D, Taylor JR (2019b) Orbitofrontal circuits control multiple reinforcement-learning processes. Neuron 103:734–746.e3. 10.1016/j.neuron.2019.05.042 31253468 PMC6893860

[B19] Hamid AA, Frank MJ, Moore CI (2021) Wave-like dopamine dynamics as a mechanism for spatiotemporal credit assignment. Cell 184:2733–2749.e16. 10.1016/j.cell.2021.03.046 33861952 PMC8122079

[B20] Hart G, Bradfield LA, Balleine BW (2018a) Prefrontal corticostriatal disconnection blocks the acquisition of goal-directed action. J Neurosci 38:1311–1322. 10.1523/JNEUROSCI.2850-17.2017 29301872 PMC6596266

[B21] Hart G, Bradfield LA, Fok SY, Chieng B, Balleine BW (2018b) The bilateral prefronto-striatal pathway is necessary for learning new goal-directed actions. Curr Biol 28:2218–2229.e17. 10.1016/j.cub.2018.05.028 30056856

[B22] Hikida T, Kimura K, Wada N, Funabiki K, Nakanishi S (2010) Distinct roles of synaptic transmission in direct and indirect striatal pathways to reward and aversive behavior. Neuron 66:896–907. 10.1016/j.neuron.2010.05.011 20620875

[B23] Igarashi KM, Ieki N, An M, Yamaguchi Y, Nagayama S, Kobayakawa K, Kobayakawa R, Tanifuji M, Sakano H, Chen WR, Mori K (2012) Parallel mitral and tufted cell pathways route distinct odor information to different targets in the olfactory cortex. J Neurosci 32:7970–7985. 10.1523/JNEUROSCI.0154-12.2012 22674272 PMC3636718

[B24] Iino Y, Sawada T, Yamaguchi K, Tajiri M, Ishii S, Kasai H, Yagishita S (2020) Dopamine D2 receptors in discrimination learning and spine enlargement. Nature 579:555–560. 10.1038/s41586-020-2115-1 32214250

[B25] Kato A, Morita K (2016) Forgetting in reinforcement learning links sustained dopamine signals to motivation. PLoS Comput Biol 12:e1005145. 10.1371/journal.pcbi.1005145 27736881 PMC5063413

[B26] Kim HF, Amita H, Hikosaka O (2017) Indirect pathway of caudal basal ganglia for rejection of valueless visual objects. Neuron 94:920–930.e3. 10.1016/j.neuron.2017.04.033 28521141 PMC5499676

[B27] Kool W, Cushman FA, Gershman SJ (2016) When does model-based control pay off? PLoS Comput Biol 12:e1005090. 10.1371/journal.pcbi.1005090 27564094 PMC5001643

[B28] Kravitz AV, Tye LD, Kreitzer AC (2012) Distinct roles for direct and indirect pathway striatal neurons in reinforcement. Nat Neurosci 15:816–818. 10.1038/nn.3100 22544310 PMC3410042

[B29] Kress GJ, Yamawaki N, Wokosin DL, Wickersham IR, Shepherd GM, Surmeier DJ (2013) Convergent cortical innervation of striatal projection neurons. Nat Neurosci 16:665–667. 10.1038/nn.3397 23666180 PMC4085670

[B30] Lee SJ, Lodder B, Chen Y, Patriarchi T, Tian L, Sabatini BL (2021) Cell-type-specific asynchronous modulation of PKA by dopamine in learning. Nature 590:451–456. 10.1038/s41586-020-03050-5 33361810 PMC7889726

[B31] Lehnert L, Littman ML (2020) Successor features combine elements of model-free and model-based reinforcement learning. J Mach Learn Res 21:1–53.34305477

[B32] Lehnert L, Tellex S, Littman ML (2017) Advantages and limitations of using successor features for transfer in reinforcement learning. arXiv 1708.00102v00101.

[B33] Lei W, Jiao Y, Del Mar N, Reiner A (2004) Evidence for differential cortical input to direct pathway versus indirect pathway striatal projection neurons in rats. J Neurosci 24:8289–8299. 10.1523/JNEUROSCI.1990-04.2004 15385612 PMC6729697

[B34] Lu J, Cheng Y, Xie X, Woodson K, Bonifacio J, Disney E, Barbee B, Wang X, Zaidi M, Wang J (2021) Whole-brain mapping of direct inputs to dopamine D1 and D2 receptor-expressing medium spiny neurons in the posterior dorsomedial striatum. eNeuro 8:ENEURO.0348-20.2020. 10.1523/ENEURO.0348-20.2020 PMC7877463 33380525

[B35] Martiros N, Kapoor V, Kim SE, Murthy VN (2022) Distinct representation of cue-outcome association by D1 and D2 neurons in the ventral striatum's olfactory tubercle. eLife 11:e75463. 35708179 10.7554/eLife.75463 PMC9203051

[B36] Matamales M, McGovern AE, Mi JD, Mazzone SB, Balleine BW, Bertran-Gonzalez J (2020) Local D2- to D1-neuron transmodulation updates goal-directed learning in the striatum. Science 367:549–555. 10.1126/science.aaz5751 32001651

[B37] Mikhael JG, Bogacz R (2016) Learning reward uncertainty in the basal ganglia. PLoS Comput Biol 12:e1005062. 10.1371/journal.pcbi.1005062 27589489 PMC5010205

[B38] Mikhael JG, Kim HR, Uchida N, Gershman SJ (2022) The role of state uncertainty in the dynamics of dopamine. Curr Biol 32:1077–1087.e9. 10.1016/j.cub.2022.01.025 35114098 PMC8930519

[B39] Momennejad I, Russek EM, Cheong JH, Botvinick MM, Daw ND, Gershman SJ (2017) The successor representation in human reinforcement learning. Nat Hum Behav 1:680–692. 10.1038/s41562-017-0180-8 31024137 PMC6941356

[B40] Morishima M, Morita K, Kubota Y, Kawaguchi Y (2011) Highly differentiated projection-specific cortical subnetworks. J Neurosci 31:10380–10391. 10.1523/JNEUROSCI.0772-11.2011 21753015 PMC6623049

[B41] Morita K (2014) Differential cortical activation of the striatal direct and indirect pathway cells: reconciling the anatomical and optogenetic results by using a computational method. J Neurophysiol 112:120–146. 10.1152/jn.00625.2013 24598515

[B42] Morita K, Kawaguchi Y (2019) A dual role hypothesis of the cortico-basal-ganglia pathways: opponency and temporal difference through dopamine and adenosine. Front Neural Circuits 12:111. 30687019 10.3389/fncir.2018.00111 PMC6338031

[B43] Morita K, Im S, Kawaguchi Y (2019) Differential striatal axonal arborizations of the intratelencephalic and pyramidal-tract neurons: analysis of the data in the MouseLight database. Front Neural Circuits 13:71. 10.3389/fncir.2019.00071 31803027 PMC6872499

[B44] Möller M, Bogacz R (2019) Learning the payoffs and costs of actions. PLoS Comput Biol 15:e1006285. 10.1371/journal.pcbi.1006285 30818357 PMC6413954

[B45] Nonomura S, Nishizawa K, Sakai Y, Kawaguchi Y, Kato S, Uchigashima M, Watanabe M, Yamanaka K, Enomoto K, Chiken S, Sano H, Soma S, Yoshida J, Samejima K, Ogawa M, Kobayashi K, Nambu A, Isomura Y, Kimura M (2018) Monitoring and updating of action selection for goal-directed behavior through the striatal direct and indirect pathways. Neuron 99:1302–1314.e5. 10.1016/j.neuron.2018.08.002 30146299

[B46] Peak J, Chieng B, Hart G, Balleine BW (2020) Striatal direct and indirect pathway neurons differentially control the encoding and updating of goal-directed learning. Elife 9:e58544. 10.7554/eLife.58544 33215609 PMC7707820

[B47] Piray P, Daw ND (2021) Linear reinforcement learning in planning, grid fields, and cognitive control. Nat Commun 12:4942. 10.1038/s41467-021-25123-3 34400622 PMC8368103

[B48] Reiner A, Hart NM, Lei W, Deng Y (2010) Corticostriatal projection neurons - dichotomous types and dichotomous functions. Front Neuroanat 4:142. 21088706 10.3389/fnana.2010.00142 PMC2982718

[B49] Russek EM, Momennejad I, Botvinick MM, Gershman SJ, Daw ND (2017) Predictive representations can link model-based reinforcement learning to model-free mechanisms. PLoS Comput Biol 13:e1005768. 10.1371/journal.pcbi.1005768 28945743 PMC5628940

[B50] Russek EM, Momennejad I, Botvinick MM, Gershman SJ, Daw ND (2021) Neural evidence for the successor representation in choice evaluation. bioRxiv. 10.1101/2021.08.29.458114.

[B51] Sakai Y, Sakai Y, Abe Y, Narumoto J, Tanaka SC (2022) Memory trace imbalance in reinforcement and punishment systems can reinforce implicit choices leading to obsessive-compulsive behavior. Cell Rep 40:111275. 10.1016/j.celrep.2022.111275 36044850

[B52] Sato R, Shimomura K, Morita K (2022) Opponent learning with different representations in the cortico-basal ganglia pathways can develop obsession-compulsion cycle. bioRxiv. 10.1101/2022.10.25.513649. PMC10306209 37319256

[B53] Stachenfeld KL, Botvinick MM, Gershman SJ (2017) The hippocampus as a predictive map. Nat Neurosci 20:1643–1653. 10.1038/nn.4650 28967910

[B54] Sutton R, Barto A (1998) Reinforcement learning. Cambridge: The MIT Press.

[B55] Tai LH, Lee AM, Benavidez N, Bonci A, Wilbrecht L (2012) Transient stimulation of distinct subpopulations of striatal neurons mimics changes in action value. Nat Neurosci 15:1281–1289. 10.1038/nn.3188 22902719 PMC3951287

[B56] Town SM, Brimijoin WO, Bizley JK (2017) Egocentric and allocentric representations in auditory cortex. PLoS Biol 15:e2001878. 10.1371/journal.pbio.2001878 28617796 PMC5472254

[B57] Wall NR, De La Parra M, Callaway EM, Kreitzer AC (2013) Differential innervation of direct- and indirect-pathway striatal projection neurons. Neuron 79:347–360. 10.1016/j.neuron.2013.05.014 23810541 PMC3729794

[B58] Wang C, Chen X, Knierim JJ (2020) Egocentric and allocentric representations of space in the rodent brain. Curr Opin Neurobiol 60:12–20. 31794917 10.1016/j.conb.2019.11.005 PMC7080648

[B59] Watabe-Uchida M, Eshel N, Uchida N (2017) Neural circuitry of reward prediction error. Annu Rev Neurosci 40:373–394. 10.1146/annurev-neuro-072116-031109 28441114 PMC6721851

[B60] Winnubst J, et al. (2019) Reconstruction of 1,000 projection neurons reveals new cell types and organization of long-range connectivity in the mouse brain. Cell 179:268–281.e13. 10.1016/j.cell.2019.07.042 31495573 PMC6754285

[B61] Yin HH, Knowlton BJ, Balleine BW (2004) Lesions of dorsolateral striatum preserve outcome expectancy but disrupt habit formation in instrumental learning. Eur J Neurosci 19:181–189. 10.1111/j.1460-9568.2004.03095.x 14750976

[B62] Yin HH, Knowlton BJ, Balleine BW (2005a) Blockade of NMDA receptors in the dorsomedial striatum prevents action-outcome learning in instrumental conditioning. Eur J Neurosci 22:505–512. 16045503 10.1111/j.1460-9568.2005.04219.x

[B63] Yin HH, Ostlund SB, Knowlton BJ, Balleine BW (2005b) The role of the dorsomedial striatum in instrumental conditioning. Eur J Neurosci 22:513–523. 16045504 10.1111/j.1460-9568.2005.04218.x

