# Supplementary figures and images for: Opponent Learning with Different Representations in the Cortico-Basal Ganglia Circuits
Source: eNeuro. 2023 Jan 25;10(1):ENEURO.0422-22.2023. doi: 10.1523/ENEURO.0422-22.2023 (PMC9884109; doi:10.1523/ENEURO.0422-22.2023)

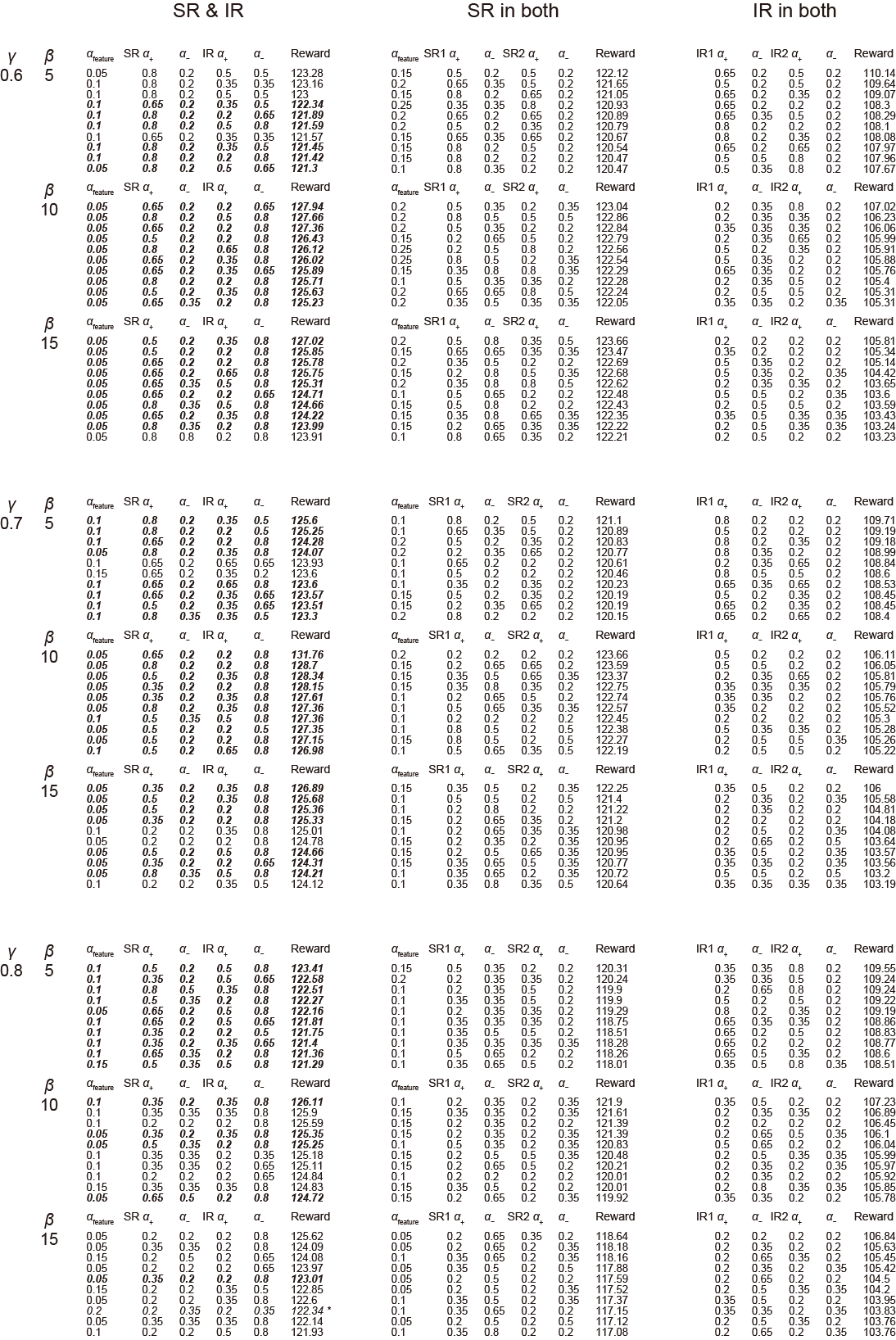
**Extended Data Figure 7-1**

Supplement: Extended Data Figure 7-1 — Performance of the models consisting of two systems in a broader parameter space. The left, middle, and right columns show the results for the model consisting of SR-based and IR-based systems, two SR-based systems, and two IR-based systems, respectively. Each subtable shows the sets of learning rate parameters that gave top ten mean performance for each set of time discount factor (γ) and inverse temperature (β; shown in the left) in each of the three models. For the model consisting of SR-based and IR-based systems, cases with a combination of appetitive (α+/α− > 1) SR-based system and aversive (α+/α− < 1) IR-based system are shown in bold italic; notably, even in all the other cases shown in the subtables except for a case shown in italic with asterisk in the right, the α+/α− ratio was higher in the SR-based system than in the IR-based system. Download Figure 7-1, DOCX file. [file enu-eN-NWR-0422-22-s03.docx]

**Extended Data Figure 8-1**


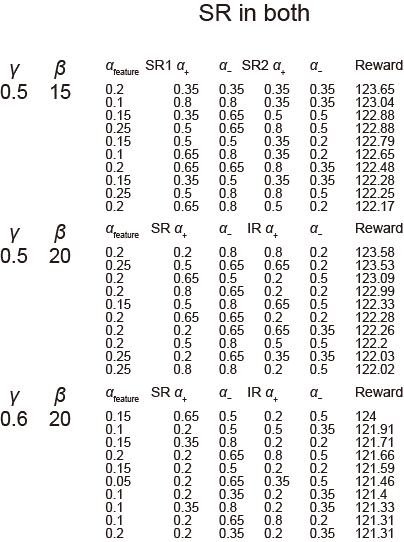

Supplement: Extended Data Figure 8-1 — Results of additional simulations for the model consisting of two SR-based systems. Each subtable shows the sets of learning rate parameters that gave top ten mean performance for each set of time discount factor (γ) and inverse temperature (β) shown in the left. Download Figure 8-1, DOCX file. [file enu-eN-NWR-0422-22-s04.docx]
